# Review of Multi-Scale Bridge Monitoring: An Information-Centric Framework Integrating Ground-Penetrating Radar, Distributed Optical-Fibre Sensing, and Satellite Remote Sensing for Bridge Digital Twins

**DOI:** 10.3390/s26185930

**Published:** 2026-09-19

**Authors:** Lilong Zou, Ying Li, Kevin Munisami, Giovanni Nico, Amir M. Alani

**Affiliations:** 1 Faculty of Engineering, Computing and the Environment, Kingston University, London KT1 1LQ, UK; l.zou@kingston.ac.uk (L.Z.); j.munisami@kingston.ac.uk (K.M.); 2 School of Computing and Mathematical Sciences, Birkbeck, University of London, London WC1E 7HX, UK; ying.li1@bbk.ac.uk; 3 Istituto per le Applicazioni del Calcolo (IAC), Consiglio Nazionale dele Ricerche (CNR), 70126 Bari, Italy; g.nico@ba.iac.cnr.it

**Keywords:** bridge monitoring, ground-penetrating radar, distributed optical-fibre sensing, satellite remote sensing, digital twin, multi-scale monitoring, multi-source information fusion

## Abstract

Bridge infrastructure worldwide is under increasing pressure from ageing assets, growing traffic demand, environmental deterioration, and the need for more effective lifecycle management. Although bridge-monitoring technologies have developed rapidly, much of the existing research has focused on individual sensing techniques, while the integration of heterogeneous information across different spatial scales has received comparatively less attention. This review takes an information-centric perspective on intelligent bridge monitoring and focuses on three representative technologies that provide complementary information at the material, structural, and network scales: Ground-Penetrating Radar (GPR) for assessing material condition, distributed optical-fibre sensing (DOFS) for monitoring structural response, and satellite remote sensing for providing network-scale information. Instead of comparing these technologies solely for their sensing principles, the review considers their complementary roles in bridge engineering and proposes a Material–Structural–Network (M–S–N) framework to organise monitoring information across scales. Recent developments in multi-source information fusion, AI-based interpretation, connected monitoring systems, autonomous monitoring, and self-evolving Digital Twins are also reviewed. Together, these advances indicate a shift from simply collecting monitoring data towards integrating information, extracting engineering knowledge, and supporting intelligent decisions. Based on this perspective, a Multi-Scale Integration Framework is proposed to illustrate how observations at different scales can be combined to provide a more complete understanding of bridge condition, structural performance, and infrastructure-network context. Future bridge monitoring should therefore move beyond isolated sensing and data collection towards multi-scale information generation and integration, supporting predictive maintenance, resilient asset management, and intelligent lifecycle decision-making across bridge networks.

## 1. Introduction

### 1.1. Challenges in Bridge Infrastructure Management

Bridges are critical components of transportation infrastructure, yet bridge networks worldwide face increasing pressures from ageing assets, growing traffic demand, environmental degradation, and limited maintenance resources. Recent infrastructure assessments have highlighted the need for more effective approaches to infrastructure management, resilience enhancement, and long-term asset stewardship [1,2].

Many existing bridges were designed and constructed several decades ago and are now approaching or exceeding their original design lives. Age-related deterioration, fatigue, corrosion, and progressive degradation of structural components can reduce structural performance while increasing maintenance requirements [3]. At the same time, increasing traffic volumes and axle loads may expose bridges to loading conditions beyond those anticipated in their original design. Environmental and climatic factors, including temperature variations, flooding, scour, and extreme weather events, can further accelerate deterioration and increase uncertainty in long-term infrastructure management [4].

Periodic visual inspection remains an essential component of bridge condition assessment, but its limited frequency, accessibility, subjectivity, and ability to detect hidden deterioration constrain comprehensive evaluation [5]. These limitations have encouraged the development of automated inspection and continuous monitoring technologies that can provide more objective and timely information on bridge condition [6]. As a result, bridge monitoring has progressively expanded from conventional inspection towards continuous sensing, remote observation, and digital infrastructure management [7].

### 1.2. From Inspection to Information-Centric Bridge Monitoring

The development of bridge monitoring has been closely linked to advances in sensing, communication, and data-processing technologies. Traditional inspection and manual surveys provide important engineering evidence, but their discontinuous nature limits the ability to capture the temporal evolution of bridge behaviour and deterioration. The emergence of structural health monitoring (SHM) introduced continuous observation through permanently installed sensors, enabling measurements of strain, vibration, displacement, and other indicators of structural response under operational conditions [3].

Subsequent advances in distributed and remote sensing have further extended the spatial coverage of bridge monitoring. Distributed optical-fibre sensing (DOFS) enables spatially continuous measurements over structural components, while satellite remote sensing provides observations over individual bridges and their surrounding environments [7]. These developments have substantially increased the volume, diversity, and spatial coverage of monitoring observations.

The resulting challenge is therefore no longer limited to acquiring measurements. More importantly, heterogeneous observations need to be interpreted and integrated within an engineering context to generate information that is useful for condition assessment and infrastructure management. This shift motivates an information-centric perspective, in which monitoring technologies are considered according to the engineering information they provide and their potential contribution to integrated bridge assessment and lifecycle management.

### 1.3. Multi-Scale Nature of Bridge Condition Information

Bridge condition cannot be fully characterised at a single spatial scale. Local deterioration, structural behaviour, and environmental or infrastructure-level influences represent different but related aspects of bridge performance. Material-scale observations can provide evidence of local physical condition; structural-scale observations describe how the bridge responds to operational and environmental loading, while network-scale observations provide broader geographical and environmental context.

These information types are complementary rather than interchangeable. An observation at one scale may indicate that a change has occurred without fully explaining its underlying cause or broader engineering significance. Reliable bridge assessment therefore requires information acquired across multiple spatial and temporal scales and interpreted in relation to one another.

This multi-scale perspective provides the conceptual basis for the Material–Structural–Network (M–S–N) framework adopted in this review. Rather than treating monitoring technologies as isolated sensing systems, the framework considers them as sources of complementary engineering information that can contribute to a more complete representation of bridge condition and performance.

### 1.4. Scope, Framework, and Contributions

A substantial body of review literature has examined individual bridge-monitoring technologies and related topics, including Ground-Penetrating Radar (GPR), SHM, distributed optical-fibre sensing, remote sensing, multisensor monitoring, and Digital Twins [8,9,10]. These studies have established important foundations for understanding individual technologies and monitoring methodologies. However, existing approaches have generally been organised around specific sensing technologies, monitoring architectures, or implementation cases. A systematic information-centric perspective linking heterogeneous observations to the different information requirements of bridge Digital Twins remains comparatively less developed.

Recent reviews further demonstrate that bridge monitoring is expanding from isolated sensing technologies towards digitalisation, Digital Twins, regional-scale monitoring, and integrated data-driven assessment. Systematic studies of bridge Digital Twins emphasise the importance of linking sensing, modelling, asset information, and management functions, while recent remote-sensing reviews highlight the complementary role of wide-area observations [11,12,13,14,15]. These developments reinforce the need for an information-centric framework that considers not only what a sensor measures, but how the resulting information can be related across spatial scales and incorporated into infrastructure decision support.

**Figure 1:**
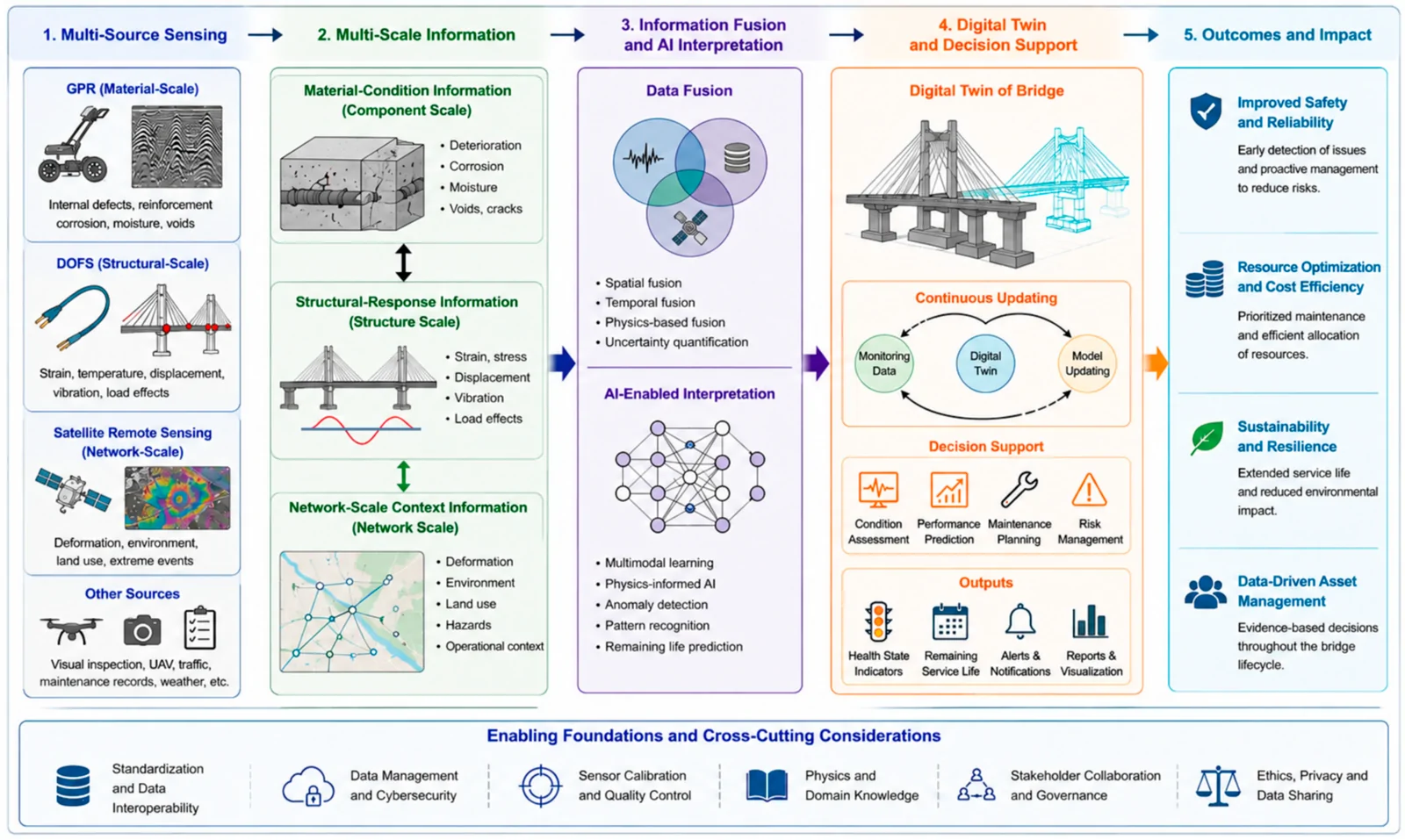
Overall research framework for multi-scale bridge monitoring and intelligent management.

To address this gap, this review examines bridge monitoring from a multi-scale information perspective. GPR, distributed optical-fibre sensing, and satellite remote sensing are considered as representative sources of complementary monitoring information rather than as competing technologies. The review focuses on how observations acquired at different scales can contribute to bridge condition assessment, structural-performance evaluation, and infrastructure-level contextual understanding. Figure 1 illustrates the Multi-Scale Bridge Monitoring Pyramid proposed in this review, which organises bridge-monitoring information into material-, structural-, and network-scale observation layers.

The main contribution of this review is the M–S–N framework, which organises bridge-monitoring information according to three complementary scales: material, structural, and network. Building on this organisation, the review examines how heterogeneous observations can be integrated, interpreted, and ultimately incorporated into bridge Digital Twins. The emphasis is therefore placed not simply on the capabilities of individual sensors, but on the transition from measurements to engineering information and, ultimately, actionable infrastructure knowledge.

The remainder of this paper is organised as follows. Section 2 describes the review methodology. Section 3 establishes the Digital Twin and multi-scale information requirements underlying the proposed framework. Section 4, Section 5 and Section 6 examine material-, structural-, and network-scale information acquisition using GPR, distributed optical-fibre sensing, and satellite remote sensing, respectively. Section 7 compares the information characteristics across spatial scales and examines their complementary roles. Section 8 develops the multi-source information integration framework for intelligent bridge monitoring. Section 9 discusses future developments, including autonomous monitoring ecosystems and self-evolving Digital Twins.

## 2. Review Methodology

A structured and comprehensive literature search was conducted to support the review of multi-scale bridge-monitoring technologies and their integration into an information-centric framework. Relevant publications were identified using Web of Science, Scopus, IEEE Xplore, and ScienceDirect, with backwards and forwards citation tracking applied to key review and seminal papers. The search combined terms describing the infrastructure domain, sensing technologies, information-processing methods, and Digital Twin applications. Representative combinations included “bridge” AND (“ground penetrating radar” OR GPR), “bridge” AND (“distributed optical fibre sensing” OR DOFS OR “distributed fiber optic”), “bridge” AND (“InSAR” OR “synthetic aperture radar” OR “satellite remote sensing”), and “bridge monitoring” AND (“multi-sensor fusion” OR “data fusion” OR “Digital Twin” OR artificial intelligence). The search covered publications from 2000 to August 2026, with particular emphasis on recent developments published between 2018 and 2026. Publications were screened according to their relevance to bridge or civil-infrastructure monitoring, contribution to sensing or information interpretation, methodological or experimental substance, and relevance to one or more spatial scales considered in the M–S–N framework. Duplicate records and publications with limited relevance to bridge condition assessment or lifecycle management were excluded. The selected literature was subsequently classified according to monitoring scale, sensing modality, engineering information, interpretation method, and potential role in bridge Digital Twin development. This information-centric classification was used to synthesise the literature rather than simply catalogue individual sensing technologies [11,16,17,18,19].

## 3. Bridge Digital Twins and Multi-Scale Information Requirements

### 3.1. Bridge Digital Twins: From Digital Representation to Intelligent Infrastructure Management

Digital Twins have emerged as an important approach for supporting the management of complex infrastructure throughout its lifecycle [20,21]. In contrast to conventional digital models that provide static or periodically updated representations, a Digital Twin maintains a continuous connection between a physical asset and its digital representation through the integration of sensing data, computational models, and operational information [22,23]. This connection enables the digital representation to reflect changes in asset condition and behaviour and provides a basis for analysis, prediction, and management decisions.

Digitalisation can be understood as a progression from a digital model to a digital shadow and ultimately to a Digital Twin. A digital model represents the physical asset without a persistent data connection, whereas a digital shadow incorporates a one-way flow of information from the physical asset to its digital representation. A Digital Twin further establishes a persistent connection through which monitoring observations can update digital representation and model-based analysis can support subsequent engineering actions. The adoption of Digital Twin concepts in civil infrastructure has therefore created opportunities for integrating monitoring, modelling, and asset management within a common digital environment [21,24].

For bridge applications, a Digital Twin typically combines the physical bridge, sensing systems, data-management infrastructure, computational models, and decision-support functions [24]. Monitoring observations are incorporated into the digital environment to support functions such as condition assessment, structural-state evaluation, anomaly detection, maintenance planning, and future-state prediction [20,23]. The effectiveness of such a system, however, depends not only on the computational models and digital infrastructure but also on whether the monitoring system can provide the engineering information required to represent the evolving state of the bridge [21,24].

Recent literature has strengthened this view by treating the bridge Digital Twin as an operational information environment rather than a static three-dimensional model. Nhamage et al. systematically reviewed strategies for combining Bridge Information Modelling, monitoring technologies, and bridge management, while Yousuf et al. examined the use of Digital Twins for bridge health assessment and management. More recent implementations have demonstrated multi-layered Digital Twin architectures, data-driven anomaly detection, and edge–cloud architectures for multi-sensor fusion [11,12,13,14,25]. Reviews of physics-informed intelligence and vibration-based SHM further indicate that model consistency, uncertainty quantification, validation, and deployment readiness remain central requirements for practical bridge Digital Twins [26,27].

This requirement shifts the focus from the Digital Twin as a digital platform to the information required to maintain its physical relevance. Bridge monitoring should therefore be considered as a process of acquiring and updating engineering information that connects observations from the physical asset with the digital representation. The central question is consequently not simply how a bridge can be monitored, but what types of information are required to describe its condition, behaviour, and surrounding context throughout its lifecycle.

### 3.2. Information Hierarchy for Bridge Digital Twins

A bridge Digital Twin requires information that can describe different aspects of an asset and its operating environment. These requirements extend beyond individual sensor measurements and include information related to the physical condition of bridge components, structural behaviour under loading, and external environmental or infrastructure influences [24]. Consequently, monitoring should be organised according to the engineering information required for assessment and decision-making rather than solely according to sensing technologies [28].

Based on this perspective, three complementary categories of engineering information are considered in this review: (a) material-condition information, describing the physical condition and deterioration of bridge components; (b) structural-response information, describing the behaviour of bridge components and structural systems under operational and environmental loading; (c) network-scale contextual information, describing environmental, geographical, and infrastructure-level conditions surrounding the bridge.

These categories represent different forms of engineering evidence. They are complementary but not interchangeable: material-condition information can indicate deterioration mechanisms, structural-response information can reveal changes in structural behaviour, and network-scale contextual information can provide the external conditions required to interpret asset behaviour. Together, they provide a more comprehensive information basis for bridge Digital Twins.

The recent literature also suggests that Digital Twin development is moving towards continuous data assimilation and decision support. Data-driven Digital Twin frameworks have combined multi-domain feature fusion with unsupervised deep learning for bridge anomaly detection, while recent bridge-management reviews have emphasised the joint role of Digital Twins, deep learning, and uncertainty-aware maintenance planning [14,29]. These studies provide direct support for viewing monitoring observations as information streams that continuously update an engineering representation of the bridge rather than as independent datasets.

#### 3.2.1. Material-Condition Information

Material-condition information describes the physical state of bridge materials and components, including deterioration mechanisms such as corrosion, cracking, delamination, moisture ingress, and material degradation. Such information provides direct evidence of localised deterioration and is therefore important for condition assessment, maintenance planning, and deterioration modelling. Previous bridge-monitoring research has demonstrated that the integration of nondestructive testing methods can provide detailed information on subsurface defects and material degradation [30]. GPR is particularly relevant to this information category because its measurements can be used to infer hidden conditions within bridge components [31]. The detailed sensing principles, measurable parameters, and engineering applications of GPR are discussed in Section 4.

#### 3.2.2. Structural-Response Information

Structural-response information characterises how bridge components and structural systems behave under operational and environmental loading. Relevant parameters include strain, displacement, vibration, acceleration, and other indicators of structural response. Such measurements are widely used in SHM to evaluate structural performance, identify abnormal behaviour, and support model validation and structural-safety assessment [32]. Distributed optical-fibre sensing provides an important source of spatially distributed structural-response information and enables long-term observation of structural behaviour [33]. Changes in measured response can provide evidence of changes in structural properties or damage evolution [34]. The principles and applications of distributed optical-fibre sensing are examined in Section 5.

#### 3.2.3. Network-Scale Contextual Information

Network-scale contextual information describes environmental and geographical processes that may influence bridge performance but extend beyond the individual structure. Relevant information includes regional ground deformation, foundation settlement, geological hazards, flooding, landslides, seismic effects, and other environmental processes. Remote sensing technologies provide an important means of acquiring such information over broad spatial and temporal scales. In particular, Persistent Scatterer Interferometric Synthetic Aperture Radar (PS-InSAR) provides a basis for long-term deformation monitoring [35], while its application to infrastructure and regional geohazard assessment has been widely demonstrated [36]. The role of satellite remote sensing in acquiring network-scale contextual information is examined in Section 6.

### 3.3. Multi-Scale Organisation of Bridge Monitoring Information

The three information categories identified above are associated with different spatial scales at which bridge-related physical processes occur and are interpreted. Bridge deterioration may originate locally within materials or components, while its effects may propagate to the structural system and may also be influenced by environmental processes occurring across much larger geographical areas. Bridge monitoring can therefore be organised as a multi-scale information-acquisition process. Accordingly, this review defines three complementary spatial levels: the material scale, structural scale, and network scale, forming the Material–Structural–Network (M–S–N) framework.

#### 3.3.1. Material Scale

The material scale represents the finest level of observation and focuses on physical processes within individual materials and structural components. Information at this scale is primarily concerned with localised deterioration and material condition. High spatial resolution and sensitivity to local anomalies are therefore important characteristics of material-scale monitoring.

#### 3.3.2. Structural Scale

The structural scale describes the behaviour of bridge components and structural systems under operational and environmental loading. Information at this scale concerns parameters such as strain, deformation, load transfer, and dynamic response, providing evidence of the mechanical state and performance of the bridge.

#### 3.3.3. Network Scale

The network scale represents the broader infrastructure and environmental system within which individual bridges operate. Information at this scale describes regional deformation, geological and climatic processes, environmental hazards, and interactions among infrastructure assets. Although such observations generally have different spatial and temporal characteristics from bridge-level measurements, they provide important contextual information for interpreting bridge behaviour.

#### 3.3.4. Multi-Scale Information Integration

The three scales should not be interpreted as independent monitoring layers. Instead, they provide complementary descriptions of bridge condition and performance. Material-scale information can provide evidence of local deterioration, structural-scale information describes the resulting or associated structural response, and network-scale information provides the broader context in which these changes occur.

The M–S–N framework therefore establishes an organisational principle for connecting different types of engineering information without assuming that one scale can replace another. The detailed characteristics of the corresponding sensing technologies are examined in Section 4, Section 5 and Section 6, while their complementary roles are compared in Section 7.

### 3.4. Scale–Information–Technology Mapping Framework

The information hierarchy and multi-scale organisation provide two complementary perspectives for structuring bridge-monitoring information. The former identifies what engineering information is required, whereas the latter identifies the spatial scale at which that information is generated and interpreted. Their combination provides a basis for relating monitoring technologies to Digital Twin information requirements.

Rather than classifying sensing technologies only according to their physical sensing principles, the present review considers them according to the engineering information they contribute to the Digital Twin. Table 1 summarises this relationship by mapping the principal information categories to their spatial scales, representative sensing technologies, and corresponding Digital Twin functions.

The mapping in Table 1 is intended as an organisational framework rather than a strict one-to-one classification. Individual sensing technologies may provide information across multiple scales, and different technologies can contribute to the same information category. Nevertheless, GPR, DOFS, and satellite remote sensing provide representative examples of three complementary information domains considered in this review.

This distinction is important for the subsequent analysis. Section 4, Section 5 and Section 6 therefore examine the three sensing technologies primarily in terms of the engineering information they can provide, their spatial and temporal characteristics, and their limitations. Section 7 then compares the resulting information across scales, while Section 8 addresses how heterogeneous observations can be aligned and integrated within an information-centric monitoring framework.

## 4. Material-Scale Information Acquisition Using GPR

### 4.1. Principles of GPR

Ground-Penetrating Radar (GPR) is a widely used nondestructive testing (NDT) technique for bridge inspection because it enables the investigation of subsurface conditions without damaging structural components. Within the framework established in Section 3, GPR is considered primarily as a source of material-scale information. Its measurements can provide evidence of reinforcement geometry, material properties, and deterioration-related conditions that are not readily accessible through surface inspection.

**Figure 2:**
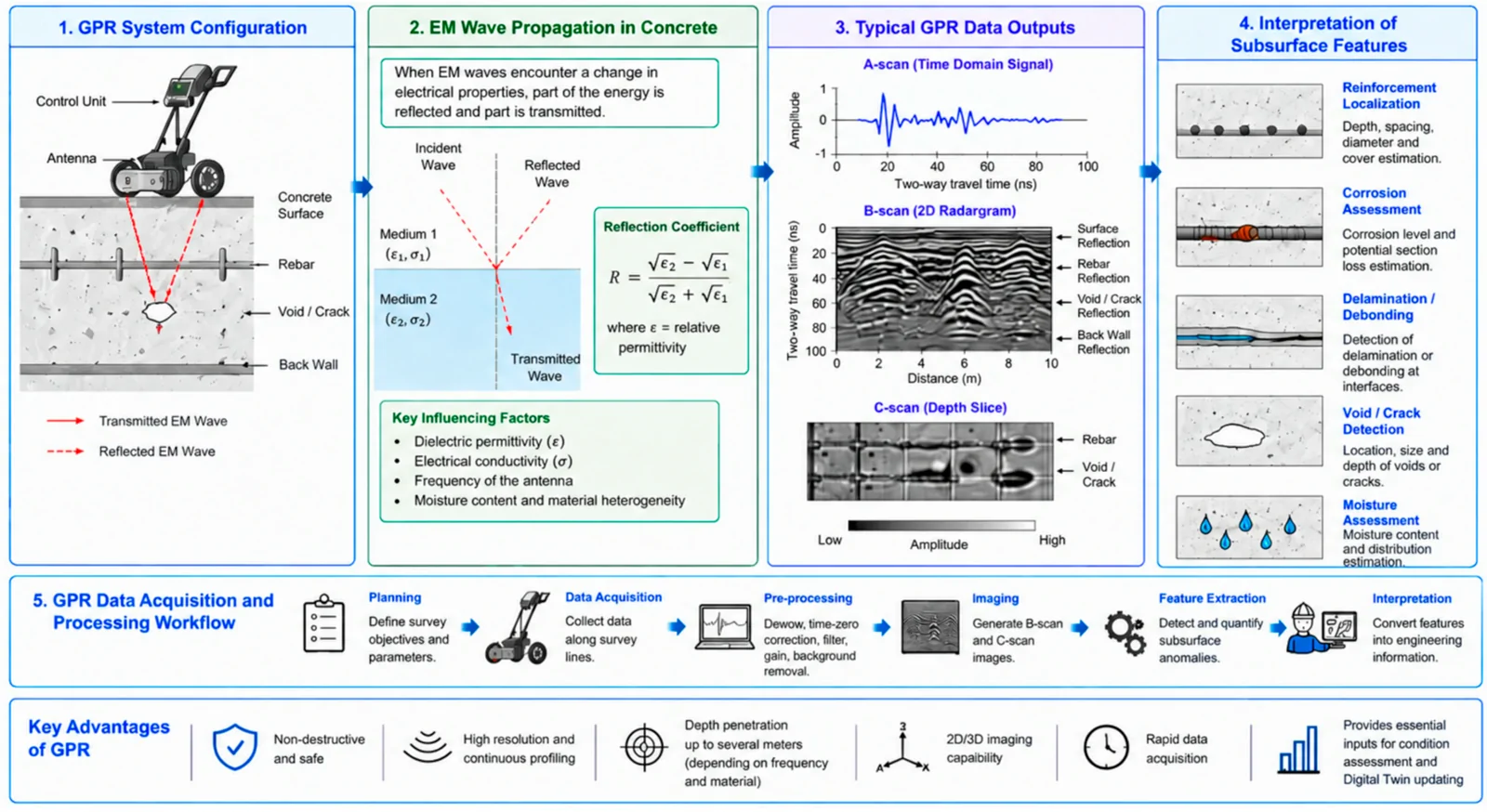
Principle of GPR for bridge inspection.

A distinction should be made between quantities directly measured by GPR and engineering conditions inferred from these measurements. GPR directly records electromagnetic responses, including travel time, reflection amplitude, phase, and signal attenuation. Engineering conditions such as corrosion, moisture accumulation, delamination, and voids are inferred from their effects on electromagnetic-wave propagation and therefore depend on physical assumptions, calibration, and independent validation. The fundamental operating mechanism of GPR is illustrated in Figure 2.

GPR transmits electromagnetic pulses into the inspected medium and records signals reflected from internal interfaces and discontinuities [37]. When an electromagnetic wave encounters a boundary between materials with different dielectric properties, part of the energy is reflected towards the receiving antenna. The resulting travel time, amplitude, phase, and spatial characteristics can then be analysed to estimate the location and properties of subsurface targets [38,39].

The relative dielectric permittivity of the inspected material is a key parameter governing electromagnetic-wave propagation. The propagation velocity can be expressed as follows:

(1) $$v=c/\sqrt{\varepsilon_{r}},$$

where v is the electromagnetic-wave velocity, c is the speed of light in free space, and $\varepsilon_{r}$ is the relative dielectric permittivity. This relationship provides the physical basis for depth estimation and dielectric-property characterisation. Because deterioration processes such as moisture ingress, chloride contamination, and changes at material interfaces can modify electromagnetic properties, GPR responses can provide indirect evidence of material degradation.

GPR performance is strongly influenced by antenna frequency. Higher frequencies generally provide better spatial resolution but shallower penetration, whereas lower frequencies provide greater penetration at reduced resolution [40]. Ground-coupled systems provide stronger electromagnetic coupling and are commonly used for detailed condition assessment, while air-coupled systems enable rapid surveys of bridge decks at traffic speed. Multi-channel and array-based systems further improve survey efficiency and spatial coverage, supporting large-area bridge inspection [41].

The principal value of GPR within the present framework is therefore not simply its ability to produce radargrams, but its ability to transform electromagnetic observations into material-condition information. The following Section 4.2 examines the principal categories of information that can be extracted from GPR measurements.

### 4.2. Material-Scale Condition Information

**Figure 3:**
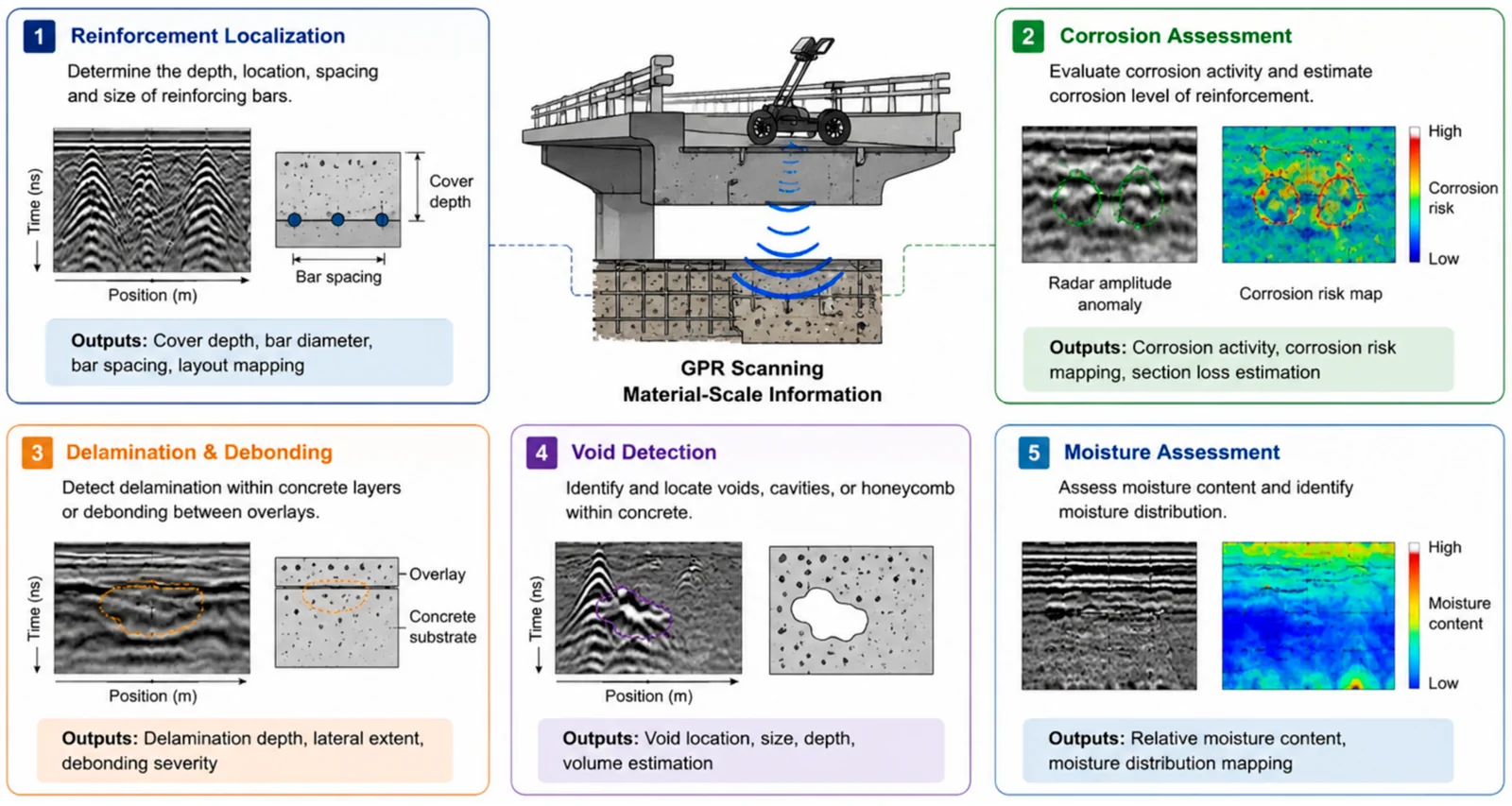
Material-scale condition information obtainable from GPR.

GPR provides several complementary forms of material-condition information. These include reinforcement geometry, corrosion-related deterioration, interface integrity, internal cavities, and material-property information. Rather than treating these as independent sensing tasks, they can be viewed as different descriptors of the physical state of bridge components. Figure 3 summarises the principal categories of material-condition information that can be obtained using GPR for bridge inspection and Digital Twin development.

#### 4.2.1. Reinforcement Localization

Bridge-deck GPR research has progressed from manual interpretation towards automated reinforcement localisation. In addition to conventional signal-processing approaches, computer-vision and deep-learning methods have been developed for automatic rebar detection, including computer-vision pipelines, deep residual networks, and pixel-level encoder–decoder architectures [42,43,44,45]. Recent work has also demonstrated hybrid approaches combining synchrosqueezed wavelet transforms, transfer learning, and optimisation for automated defect identification in real bridge-deck surveys [46].

Reinforcement localization is one of the most established applications of GPR in bridge inspection. Accurate information on reinforcement position, spacing, and concrete cover is important because reinforcement geometry affects electromagnetic-wave propagation and provides a reference for interpreting subsequent deterioration indicators [47,48]. Steel reinforcement produces strong electromagnetic contrasts with the surrounding concrete. Under common-offset configurations, reflections from reinforcing bars typically produce characteristic hyperbolic signatures in B-scan data. Their geometry depends on reinforcement depth, antenna frequency, acquisition configuration, and the dielectric properties of the surrounding material. Reinforcement position and cover depth can therefore be estimated from reflection geometry and travel-time characteristics.

Early applications relied mainly on manual radargram interpretation and geometric fitting. As bridge inspection programmes expanded, semi-automated approaches based on signal processing, hyperbola detection, and geometric constraints were developed to reduce operator dependence and improve consistency [49]. Dense-array and multi-channel GPR systems have further increased the efficiency of reinforcement mapping over large bridge decks [50,51]. Despite these developments, reinforcement localization remains sensitive to dense reinforcement layouts, overlapping reflections, heterogeneous concrete, signal attenuation, dielectric-permittivity assumptions, and acquisition geometry. Independent verification is therefore important when geometric information is subsequently used for quantitative deterioration assessment.

#### 4.2.2. Corrosion Assessment

Corrosion assessment has become a particularly active area of bridge-deck GPR research. A systematic review by Faris et al. synthesised the physical, environmental, and technical factors affecting GPR-based corrosion interpretation, while subsequent work demonstrated automated rebar recognition and corrosion mapping from bridge-deck GPR data [52,53]. These studies indicate that GPR amplitude and waveform characteristics can provide spatially distributed indicators of reinforcement corrosion, but also emphasise the need to account for moisture, chloride exposure, signal attenuation, and acquisition conditions when converting GPR measurements into condition information.

Reinforcement corrosion is a major deterioration mechanism in reinforced-concrete bridges because corrosion can reduce the capacity of reinforcing steel and contribute to cracking, delamination, and progressive concrete degradation. GPR does not directly measure electrochemical corrosion activity. Instead, it detects electromagnetic changes associated with corrosion-related changes in the surrounding material and steel–concrete interface. Chloride contamination, moisture accumulation, increased electrical conductivity, cracking, and changes in the steel–concrete interface can influence electromagnetic propagation. These effects may appear as variations in reflection amplitude, attenuation, travel time, or waveform characteristics. Early studies therefore established relationships between the attenuation of reinforcing-bar reflections and bridge-deck deterioration [54].

Subsequent research introduced corrections for reinforcement depth and signal attenuation, together with dielectric-property estimation, depth normalisation, and statistical analysis, to reduce the effects of acquisition geometry and material variability. These developments have improved the consistency of GPR-based deterioration assessment and enabled larger-area condition mapping. However, corrosion-related GPR responses remain non-unique. Moisture content, chloride concentration, concrete heterogeneity, reinforcement geometry, and construction variability can produce overlapping electromagnetic effects [55]. Consequently, GPR-derived corrosion indicators should be interpreted as indirect evidence rather than definitive measurements of corrosion. Quantitative assessment requires calibration and, where possible, independent measurements or complementary NDT techniques.

#### 4.2.3. Delamination and Debonding Detection

Automated delamination detection has also expanded rapidly. Recent studies have applied 1D-CNNs and hybrid deep-learning frameworks to bridge-deck GPR data, demonstrating that contextual information and transfer learning can improve classification of delaminated regions [46,56]. A recent multi-modal study further combined GPR with infrared thermography through physics-enhanced feature fusion, showing the potential of heterogeneous NDE data to improve generalisation beyond single-modality interpretation [57].

Delamination and debonding represent deterioration of material interfaces and are particularly relevant to layered bridge decks and composite structures. They may result from reinforcement corrosion, repeated loading, construction deficiencies, environmental exposure, or long-term material degradation [58]. GPR is sensitive to changes in electromagnetic properties at material interfaces. Interface separation can modify local impedance and produce changes in reflection amplitude, waveform characteristics, and propagation behaviour. Conventional assessment commonly relies on anomalous reflections in B-scan images, but interpretation can be affected by reinforcement interference, moisture, defect thickness, and signal attenuation.

Thin or discontinuous interface defects are particularly difficult to identify when their dimensions are small relative to the radar wavelength. This limitation has motivated the use of acquisition and processing strategies that exploit electromagnetic-wave propagation beyond conventional reflection amplitude. Multi-static acquisition and Common Mid-Point (CMP) analysis, for example, can provide additional information from lateral-wave propagation and velocity characteristics [37]. Time–frequency representations and wavelet-entropy features have also been investigated to improve the discrimination of weak interface responses [51]. The resulting development represents a shift from image-based defect identification towards more physically informed characterisation of interface integrity. Nevertheless, GPR indications of delamination or debonding should still be regarded as condition evidence requiring appropriate validation rather than as unique defect identification.

#### 4.2.4. Moisture Assessment

Moisture remains an important confounding factor and an engineering target in GPR-based bridge assessment. Bridge-deck studies have shown that dielectric properties and electromagnetic attenuation are strongly influenced by water content, which can affect both cover-depth estimation and deterioration interpretation [17,59,60,61]. Consequently, recent GPR research increasingly treats moisture and environmental conditions as contextual variables that should be incorporated into automated interpretation rather than ignored during defect classification.

Moisture is an important material property in GPR-based bridge assessment because water strongly affects electromagnetic-wave propagation and can influence the interpretation of other GPR observations. Moisture is also closely associated with reinforcement corrosion, freeze–thaw deterioration, and long-term material degradation. The sensitivity of GPR to moisture originates from the large difference between the dielectric properties of water and those of most construction materials. Variations in water content can therefore modify propagation velocity, travel time, reflection characteristics, and signal attenuation. These relationships provide the basis for estimating moisture conditions and characterising dielectric properties [62].

Research has established relationships between GPR-derived electromagnetic parameters and subsurface water content, with dielectric-property analysis providing a means of characterising moisture distribution [63]. More recent approaches have focused on improving dielectric-constant estimation and quantitative moisture retrieval [64]. An important characteristic of moisture information is that it also affects the interpretation of other material-condition indicators. Changes in water content can influence the assessment of corrosion, interface integrity, and internal anomalies. Moisture estimation is therefore relevant both as an independent condition indicator and as supporting information for interpreting other GPR responses. Quantitative moisture estimation remains affected by material composition, pore structure, temperature, chloride concentration, and material heterogeneity. Consequently, reliable moisture characterisation requires appropriate calibration and consideration of the interacting factors that influence electromagnetic responses [65].

Table 2 summarises the five principal categories of material-conditioned information discussed in Section 4.2.4, together with their representative engineering targets, physical interpretations, characteristic GPR signatures, conventional interpretation strategies, and emerging research directions. Rather than replacing conventional defect-oriented descriptions, this classification provides a unified perspective for understanding the complementary roles of GPR in material-scale bridge condition assessment.

An important limitation is that GPR responses are generally non-unique. Similar electromagnetic signatures may arise from different combinations of moisture, chloride contamination, material heterogeneity, reinforcement geometry, interface conditions, and other factors. As a result, both false-positive and false-negative interpretations are possible, particularly for subtle deterioration. The reliability of GPR-derived information therefore depends on acquisition configuration, dielectric assumptions, signal processing, calibration, and independent validation. Bridge-specific ground truth, complementary NDT methods, and repeated measurements are particularly important when GPR observations are used for quantitative condition assessment or Digital Twin updating.

### 4.3. AI-Assisted Information Extraction

Traditional GPR interpretation relied on manual examination of radargrams and engineering experience. Hyperbolic reflections, amplitude variations, waveform characteristics, and other patterns were interpreted to identify reinforcement and potential deterioration. Although this approach remains useful, it is labour-intensive and difficult to standardise across large bridge inventories.

Signal-processing and handcrafted-feature approaches subsequently provided more structured representations of GPR observations. Time-domain and frequency-domain features, texture descriptors, wavelet representations, and entropy-based measures were incorporated into automated workflows. Conventional machine-learning algorithms, including Support Vector Machines, Artificial Neural Networks, Decision Trees, Random Forests, and clustering methods, were then applied to bridge-inspection tasks.

The principal significance of this development was the transition from manual interpretation towards automated information extraction. However, model performance remained strongly dependent on feature design, preprocessing, and domain expertise, limiting transferability across different bridge structures, material conditions, and acquisition systems.

#### 4.3.1. Deep Learning for Automated Material-Condition Information Extraction

The recent expansion of bridge-specific GPR datasets has enabled more representative deep-learning studies. Large field datasets collected from multiple bridge decks have been used to train object-detection models for rebar recognition, while other studies have explored pixel-level classification and hybrid time–frequency/deep-learning approaches [42,43,44,45,46,56,66]. This trend is important for the present framework because automated interpretation converts dense GPR measurements into structured engineering information such as rebar location, cover depth, corrosion indicators, and delamination probability [67].

Deep learning has reduced the dependence on manually engineered features by learning hierarchical representations directly from GPR data [68,69,70,71]. Convolutional neural networks have been widely investigated for reinforcement localisation, where the recognition of hyperbolic signatures can be automated over large bridge-deck datasets. Deep-learning methods have subsequently been applied to corrosion-related deterioration, delamination and debonding, internal anomalies, and moisture-related condition assessment. Their ability to learn spatial and contextual features is particularly useful for electromagnetic responses that cannot be reliably separated using simple thresholds or manually selected features.

Another important development is the use of domain-specific data representations. Rather than applying computer-vision models directly to raw radargrams, researchers have investigated signal transformations, multi-domain representations, and physics-aware preprocessing to improve the discriminative characteristics of GPR observations. The research direction is also moving from individual defect-recognition tasks towards the extraction of multiple categories of material-condition information. This shift is important for the information-centric framework because the objective is ultimately to generate a structured representation of material condition rather than a collection of independent defect labels. Despite these advances, practical deployment remains constrained by limited labelled datasets, differences among sensing systems and acquisition conditions, and the lack of standardised bridge-GPR benchmarks [72,73].

#### 4.3.2. Emerging AI Paradigms for Material-Condition Information Extraction

An additional direction is the integration of physics and heterogeneous sensing information into GPR interpretation. Recent bridge-deck studies have combined GPR with complementary NDE modalities, while broader physics-informed and hybrid-learning approaches provide a pathway for constraining data-driven models with electromagnetic or structural knowledge [26,57]. Such approaches are particularly relevant where field-labelled GPR datasets are limited or where changes in acquisition conditions reduce the transferability of purely data-driven models.

The limitations of conventional deep-learning approaches have motivated several emerging directions. Transformer-based architectures can capture long-range dependencies and contextual relationships in large and complex datasets and have therefore attracted increasing attention for GPR interpretation [74,75]. Physics-informed learning provides another important direction. GPR observations are governed by electromagnetic-wave propagation and are influenced by antenna characteristics, material properties, acquisition geometry, and subsurface interfaces. Incorporating such physical constraints into learning models can improve robustness and interpretability and reduce reliance on purely empirical relationships [76].

Simulation-assisted learning offers a complementary solution to the limited availability of labelled field data. Electromagnetic modelling based on methods such as finite-difference time-domain (FDTD) simulation can generate physically consistent synthetic observations for model development and evaluation [77]. Advances in sensing-system design are also closely related to AI-based interpretation. Multi-channel arrays, multi-static configurations, dual-polarised measurements, and three-dimensional acquisition can provide richer observations for subsequent information extraction. The emerging trend is therefore towards the joint development of sensing, electromagnetic modelling, data representation, and intelligent interpretation, rather than treating the sensing system and AI algorithm as independent components.

### 4.4. Interpretation Challenges

Despite substantial progress, several challenges limit the use of GPR as a reliable source of quantitative material-condition information.

First, there is a continuing gap between anomaly detection and quantitative condition characterisation. GPR can identify characteristic responses associated with reinforcement, deterioration, interfaces, and moisture, but estimating deterioration severity, material properties, geometric dimensions, and associated uncertainty remains more difficult. Future development therefore needs to emphasise quantitative inversion and parameter estimation rather than binary defect classification.

Second, the physical non-uniqueness of electromagnetic responses limits the reliability of purely data-driven interpretation. Similar radar responses can result from different combinations of material properties, moisture, chloride contamination, reinforcement geometry, and acquisition conditions. Physics-informed modelling and independent validation are therefore important for improving engineering reliability.

Third, the quality and diversity of the acquired observations remain fundamental limitations. Multi-channel, multi-static, dual-polarised, and three-dimensional systems can provide richer information, but the resulting increase in data volume also increases requirements for processing, calibration, and data management.

Fourth, the lack of large and standardised benchmark datasets limits objective comparison and model transferability. Existing studies often rely on laboratory experiments, numerical simulations, or locally collected datasets with different sensing systems and annotation strategies. More representative datasets covering different bridge types, material conditions, environmental conditions, and acquisition configurations are needed to support reproducible evaluation.

Finally, GPR-derived material information must ultimately be interpreted together with information acquired at other spatial scales. Material-condition information alone cannot fully describe structural behaviour or environmental context. The next step is therefore not simply to improve GPR-based defect detection, but to generate reliable material-condition descriptors that can be integrated with structural-response and network-scale information.

### 4.5. Role of GPR in the Multi-Scale Bridge Monitoring Framework

Within the M–S–N framework, GPR provides the principal source of material-condition information. Its main contribution is the characterisation of subsurface geometry, deterioration, interface integrity, and material properties that may not be observable through conventional surface inspection. The progression of GPR research from radargram interpretation to quantitative and AI-assisted information extraction is therefore consistent with the broader information-centric perspective adopted in this review. The objective is to transform electromagnetic observations into physically meaningful descriptors of material condition that can subsequently be combined with structural-response and network-scale information. The following Section 5 extends this perspective to the structural scale, where distributed optical-fibre sensing provides spatially resolved observations of bridge response under operational and environmental loading.

## 5. Optical-Fibre Sensing for Structural-Response Monitoring

### 5.1. Classification of Optical-Fibre Sensing Technologies

Optical-fibre sensing (OFS) provides a flexible approach to monitoring the response of bridge structures because optical fibres can be used to measure mechanical and environmental quantities with high spatial resolution and over long sensing distances. In this review, Fibre Bragg Grating (FBG) sensors are considered discrete optical-fibre sensors, whereas Brillouin-based sensing, Rayleigh backscattering/Optical Frequency Domain Reflectometry (OFDR), and distributed acoustic sensing (DAS) are considered distributed sensing techniques. Accordingly, OFS is used as the umbrella term, while DOFS refers specifically to distributed optical-fibre sensing.

The sensing principle depends on changes in the optical properties of light propagating through an optical fibre. Mechanical strain, temperature variation, and dynamic disturbance can modify the characteristics of the backscattered or reflected optical signal. These changes can be analysed to estimate the corresponding physical quantities along the sensing fibre. Early studies demonstrated the feasibility of fibre-optic sensing for structural monitoring [78], while subsequent research established the underlying sensing principles and extended fibre-optic techniques to large civil-engineering structures [79,80].

The main advantage of distributed sensing is not simply the increase in the number of measurement points, but the ability to preserve the spatial continuity of structural-response information. Instead of obtaining measurements only at selected locations, distributed fibre systems can provide response profiles along structural components. Such measurements can reveal strain redistribution, deformation compatibility, local response concentrations, and changes in structural behaviour under operational and environmental loading.

Different OFS technologies provide different combinations of spatial resolution, sensing length, measurement bandwidth, and response type. FBG sensors provide accurate measurements at discrete locations and are well suited to monitoring critical structural components. Brillouin-based systems provide distributed strain and temperature measurements over long sensing lengths. Rayleigh-based techniques, particularly OFDR, offer very high spatial resolution for localised deformation and crack-related responses. DAS extends optical-fibre monitoring into the dynamic domain by providing spatially distributed measurements associated with vibration and wave propagation.

**Figure 4:**
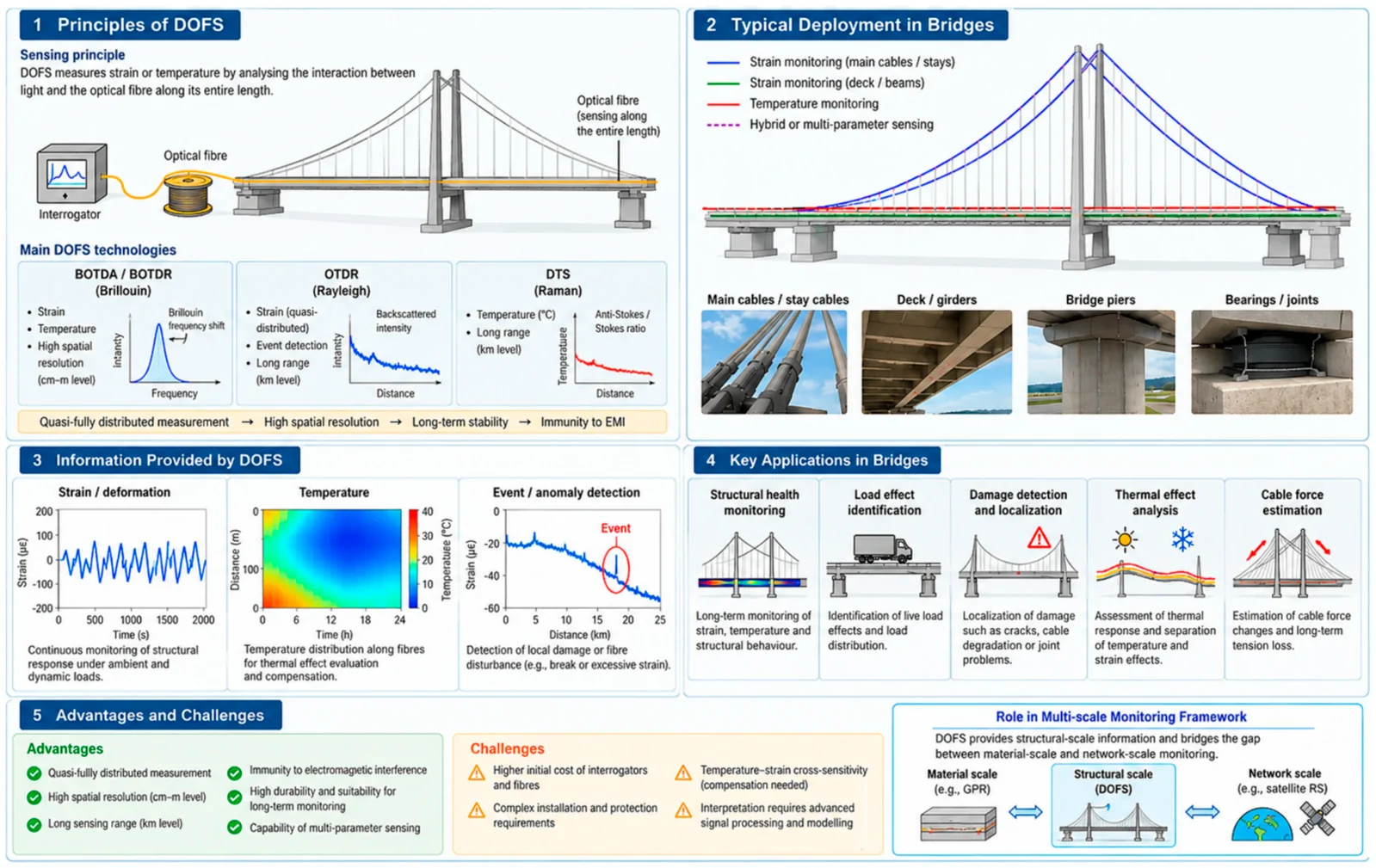
Structural-scale information acquisition using DOFS.

An important distinction should be made between quantities measured directly by optical-fibre systems and engineering information inferred from those measurements. Strain, temperature, and optical-phase changes are measurement outputs, whereas deformation, crack development, stiffness variation, load redistribution, and structural condition are engineering interpretations derived using sensor models and structural analysis. The reliability of these interpretations therefore depends on sensor installation, strain transfer, temperature compensation, calibration, and the structural context in which the measurements are evaluated. Figure 4 summarises the principal categories of DOFS for bridge inspection and Digital Twin development. The following Section 5.2 examines the principal categories of structural-response information provided by OFS and the corresponding sensing technologies.

### 5.2. Structural-Response Information Provided by OFS

Structural-response information describes how bridge components and structural systems behave under operational and environmental loading. Unlike material-condition information obtained from techniques such as GPR, structural-response information reflects mechanical behaviour through measurements such as strain, deformation, temperature-related response, and dynamic characteristics.

OFS provides several complementary forms of structural-response information. The appropriate sensing technology depends on the required spatial resolution, sensing distance, temporal resolution, and structural application. Rather than treating FBG, Brillouin, Rayleigh/OFDR, and DAS as competing technologies, they can be viewed as complementary approaches for observing structural behaviour at different spatial and temporal scales.

#### 5.2.1. FBG: Discrete Optical-Fibre Sensing

FBG sensing has a long history in bridge monitoring, including applications to the Tsing Ma Bridge, composite bridges, and long-term monitoring of concrete bridges [81,82,83]. These studies established the value of fibre-optic measurements for strain, displacement, load, and long-term structural response. More recent reviews confirm that FBG technology remains relevant for civil SHM, particularly where discrete high-accuracy measurements and embedded sensing are required [84,85].

Although FBG sensors are not strictly distributed sensing technologies, they remain one of the most widely adopted optical-fibre techniques in bridge structural health monitoring because of their high measurement accuracy, rapid response, multiplexing capability, and long-term stability [55,56]. Their technological maturity has supported applications ranging from laboratory investigations to full-scale bridge monitoring.

The principal contribution of FBG sensing is the measurement of local structural response at selected locations. Strain, deformation, and temperature measurements can be obtained from embedded or surface-mounted sensors and subsequently used to evaluate structural behaviour under operational and environmental loading. Such measurements provide information for structural-state assessment, performance evaluation, and validation of analytical models [86,87].

Applications of FBG sensing have progressively expanded to concrete, steel, and composite bridges. Long-term monitoring studies have demonstrated the feasibility of FBG systems under realistic service conditions [88], while both embedded and surface-mounted configurations have been investigated for structural monitoring [89]. Measurements obtained at carefully selected locations can reveal local strain concentrations, load redistribution, deformation compatibility, and changes in response under varying operational conditions. These observations can support proof-load testing, model validation, and the evaluation of strengthening measures [90].

FBG sensing has also been applied to reinforced-concrete structures, where local strain measurements can assist in identifying crack initiation and tracking structural response during progressive deterioration. However, the discrete nature of FBG sensing limits spatial coverage. Structural responses occurring between instrumented locations may remain undetected, and monitoring performance therefore depends strongly on sensor-placement strategies. Temperature effects and environmental influences also require appropriate compensation when long-term measurements are interpreted. These limitations provide a motivation for distributed optical-fibre techniques, which can extend structural-response measurements from selected locations to continuous or quasi-continuous sensing regions.

#### 5.2.2. Brillouin-Based Distributed Sensing

Brillouin-based distributed sensing extends this capability from discrete points to long measurement lengths. Field studies have demonstrated distributed strain measurements on reinforced-concrete bridges, while integrated inverse-analysis approaches have used distributed fibre measurements to reconstruct deformation and stress [91,92,93]. Recent bridge monitoring using specially installed distributed fibres has further demonstrated three-dimensional deformation estimation and rapid post-earthquake assessment over approximately kilometre-scale bridge lengths [94,95].

Brillouin-based distributed optical-fibre sensing, including Brillouin Optical Time Domain Analysis (BOTDA) and Brillouin Optical Time Domain Reflectometry (BOTDR), enables distributed measurements of strain and temperature over optical fibres with sensing lengths extending to the kilometre scale [96,97]. Compared with discrete instrumentation, these systems provide substantially greater spatial coverage and enable structural-response profiles to be obtained over large bridge components [98].

The main engineering value of Brillouin sensing lies in its ability to characterise the spatial distribution of structural response. Distributed strain profiles can reveal load transfer, deformation compatibility, strain redistribution, and response evolution along structural members. Such information is particularly relevant to long-span bridges, prestressed components, and structures in which distributed changes in response may be difficult to capture using discrete sensors.

The spatial continuity of the measured response also provides opportunities for structural-model validation. Measured strain distributions can be compared with numerical predictions to assess model assumptions, calibrate structural parameters, and identify deviations from expected behaviour. This approach provides a broader basis for structural-state assessment than threshold-based evaluation of individual measurement points.

Brillouin sensing has nevertheless practical limitations. Spatial resolution, sensor installation, strain-transfer characteristics, temperature compensation, and long-term environmental effects can all influence the reliability of the measured response. Appropriate interpretation therefore requires consideration of both sensing performance and structural mechanics. The principal value of Brillouin-based sensing is consequently its ability to convert distributed optical measurements into spatially continuous evidence of structural behaviour.

#### 5.2.3. Rayleigh Backscattering and OFDR

Rayleigh-based distributed sensing and high-resolution optical-frequency-domain techniques provide complementary capabilities for localised deformation and crack-related observations. Their principal advantage within the M–S–N framework is the ability to generate spatially dense structural-response information, reducing the dependence on a small number of point sensors and supporting more detailed comparison with structural models [84,92].

Rayleigh backscattering-based sensing, particularly Optical Frequency Domain Reflectometry (OFDR), provides distributed measurements with very high spatial resolution. Compared with Brillouin-based systems, which are generally more suitable for long sensing distances, OFDR is particularly useful for detailed investigation of localised deformation over relatively short sensing lengths. It has therefore attracted increasing attention for component-level assessment and damage characterisation [99].

A major advantage of Rayleigh-OFDR sensing is its ability to identify local strain concentrations associated with cracking. With an appropriate strain-transfer model, the measured strain distribution may also be used to estimate crack location and, under suitable conditions, crack opening. The reliability of such estimates depends on factors including fibre bonding, cable construction, gauge length, strain range, and the ability of the sensing system to maintain adequate coupling across the crack.

Near-continuous strain measurements provide information that cannot be obtained readily from isolated sensors. Spatial strain gradients, local response concentrations, and deformation compatibility can be analysed to distinguish local anomalies from broader structural behaviour. Such information is relevant to crack localisation, verification of repair effectiveness, assessment of strengthening measures, and numerical-model validation. Distributed fibre-optic measurements can therefore contribute to performance-oriented bridge-monitoring frameworks [100].

Recent developments have increasingly combined OFDR measurements with numerical modelling, image-based inspection, and advanced data-analysis methods. The emphasis is gradually shifting from achieving higher sensing resolution alone towards extracting reliable engineering information from distributed measurements. Within the broader OFS framework, Rayleigh-OFDR complements FBG and Brillouin-based sensing by providing high-resolution information on local deformation and damage evolution.

#### 5.2.4. Distributed Acoustic Sensing (DAS)

DAS extends distributed optical sensing into the dynamic domain. Recent bridge studies have demonstrated dynamic monitoring using distributed acoustic sensing on railway and urban bridge networks, including extraction of vibration frequencies, damping, modal shapes, and dynamic strain responses [18,101,102]. These developments suggest that optical fibre systems can provide both quasi-static structural-response information and distributed dynamic information, although interrogator bandwidth, gauge length, signal attenuation, and data volume remain practical constraints.

Distributed acoustic sensing (DAS) extends optical-fibre monitoring from predominantly static and quasi-static measurements towards a dynamic structural response. By measuring distributed optical-phase changes associated with dynamic strain or strain-rate responses, DAS can capture bridge behaviour associated with traffic loading, train passages, wind excitation, and other dynamic actions [103].

A key advantage of DAS is its ability to provide spatially distributed measurements of dynamic response over long sensing lengths. Dense measurements along the fibre can reveal vibration patterns, wave-propagation characteristics, and spatial variations in dynamic response. Compared with conventional vibration-monitoring systems based on discrete accelerometers or geophones, DAS provides distributed measurements along the sensing fibre. DAS does not directly measure acceleration; vibration characteristics and modal properties are derived subsequently through signal processing and system-identification procedures.

The resulting dynamic information can be used to investigate modal behaviour, wave propagation, and changes in structural response. Variations in modal characteristics, vibration amplitudes, and spatial response distributions may indicate changes in structural behaviour, altered boundary conditions, or evolving damage mechanisms. Dynamic measurements therefore provide complementary information to static and quasi-static strain measurements and can be compared with structural-model predictions.

The practical performance of DAS depends on cable coupling, fibre orientation, gauge length, measurement noise, and the characteristics of the interrogator. The use of existing telecommunication fibre networks is also being investigated as a means of increasing monitoring coverage and reducing deployment requirements. However, such systems do not necessarily eliminate the need for dedicated sensing fibres where specific structural configurations or measurement characteristics are required.

Overall, DAS provides a complementary dynamic dimension to OFS-based structural monitoring. Its value lies not simply in the continuous acquisition of vibration data, but in the extraction of spatially distributed dynamic-response information that can support structural assessment.

### 5.3. AI-Assisted Structural-Response Information Interpretation

AI-assisted interpretation is becoming increasingly relevant as distributed optical-fibre systems generate large volumes of spatially and temporally dense data. Recent reviews of fibre-optic sensing combined with deep learning and AI-assisted fibre sensing highlight automated anomaly detection, classification, and predictive maintenance as emerging directions [104]. For bridge Digital Twins, the value of these approaches lies not simply in classifying raw fibre signals, but in transforming distributed measurements into interpretable structural-response indicators that can be assimilated into engineering models.

The increasing deployment of OFS has substantially increased both the spatial density and temporal volume of bridge-monitoring data. This development shifts an important part of the monitoring challenge from data acquisition towards interpretation. Large, distributed datasets must be transformed into structural-response information that is relevant to performance assessment, anomaly identification, and lifecycle management.

A fundamental distinction should be maintained between measured response and structural condition. Distributed strain, temperature, and dynamic-response measurements describe how a bridge behaves under particular loading and environmental conditions, but they do not directly identify deterioration mechanisms or determine structural safety. Similar response patterns may result from traffic loading, thermal effects, boundary-condition changes, construction characteristics, or genuine structural deterioration. Consequently, intelligent interpretation must account for the structural and operational context of the observations.

Traditional interpretation relies on structural mechanics, numerical modelling, baseline measurements, and engineering judgement. Distributed measurements can be compared with analytical or finite-element predictions to identify abnormal response patterns and evaluate structural performance. These approaches remain important because they provide the physical basis for interpreting changes in measured response.

The increasing volume of OFS data has nevertheless stimulated the use of machine-learning methods for anomaly detection, feature extraction, pattern recognition, and structural-condition classification. Post-processing methods developed specifically for distributed optical-fibre measurements have highlighted the importance of signal quality, feature representation, and engineering knowledge in obtaining reliable structural information [105]. Rather than replacing mechanics-based interpretation, AI can assist in identifying response patterns that require further engineering investigation.

Future intelligent interpretation is expected to increasingly combine OFS measurements with numerical models, inspection records, maintenance histories, environmental observations, and other monitoring sources. Explainable learning, uncertainty-aware inference, and physics-informed modelling are particularly relevant because the objective is not simply to classify response patterns but to establish interpretable relationships between measurements and structural behaviour.

Accordingly, AI-assisted OFS interpretation should be viewed as an information-extraction process. Its purpose is to convert large volumes of distributed measurements into reliable structural-response descriptors that can support engineering assessment and subsequent multi-source information integration.

### 5.4. Current Challenges and Future Perspectives

Recent distributed sensing studies also highlight several issues that limit direct transfer between bridges. Strain-transfer behaviour, installation configuration, gauge length, temperature sensitivity, interrogation technology, and environmental exposure can alter the measured response. Long-term railway and bridge deployments demonstrate the importance of validating distributed measurements against conventional sensors or structural models [92,94,106]. These findings support the use of uncertainty-aware interpretation and model-based validation when optical-fibre observations are incorporated into a bridge Digital Twin.

Despite significant advances in optical-fibre sensing, several challenges remain before distributed response measurements can be converted consistently into reliable engineering evidence for bridge assessment.

First, the relationship between structural response and structural condition is inherently non-unique. Changes in strain, deformation, or vibration may result from operational loading, temperature, environmental effects, boundary-condition changes, or deterioration. Consequently, abnormal response does not necessarily provide direct evidence of damage, and structural interpretation requires appropriate engineering context.

Second, measurement reliability remains strongly dependent on installation and sensor–structure interaction. Fibre bonding, cable construction, strain transfer, temperature compensation, environmental exposure, and long-term durability can all influence the measured response. These effects introduce uncertainties that may propagate into subsequent structural assessment [107].

Third, the large volume of continuously acquired distributed data creates challenges for storage, processing, feature extraction, and long-term interpretation. Increasing measurement density does not automatically lead to improved engineering understanding. Effective monitoring therefore requires methods that identify the response characteristics most relevant to structural assessment while retaining the spatial information provided by distributed sensing.

Fourth, the transferability of intelligent interpretation models remains limited. Differences in bridge type, structural configuration, sensing fibre, installation method, interrogator, environmental conditions, and operational loading can alter the measured response. Models trained under one set of conditions may therefore not generalise directly to other structures.

Finally, structural-response information alone cannot explain all aspects of bridge condition. Response observations should be interpreted together with material-condition information, environmental context, structural models, and inspection evidence. Improving this integration is therefore essential for converting distributed sensing measurements into robust information for bridge Digital Twins.

### 5.5. Role of OFS in Bridge Digital Twins

Within the framework established in Section 3, OFS provides structural-response information that complements the material-condition information obtained from GPR. Its principal contribution is the spatially resolved observation of how bridge components and structural systems respond to operational and environmental loading.

Different OFS technologies provide complementary response information. FBG sensors provide accurate measurements at selected locations, Brillouin-based systems provide long-range distributed strain and temperature information, Rayleigh-OFDR provides high-resolution observations of local deformation, and DAS extends monitoring into the dynamic domain. Together, these approaches provide multiple views of structural behaviour across different spatial and temporal scales.

The main engineering value of OFS therefore lies in transforming optical measurements into reliable structural-response information. Such information can support structural-state assessment, model validation, performance evaluation, anomaly identification, and long-term monitoring. However, OFS does not directly identify the underlying material deterioration responsible for an observed response. Its interpretation is therefore strengthened when structural-response information is considered together with material-condition observations and appropriate engineering models.

This complementary relationship provides the basis for the next stage of the multi-scale monitoring framework. While GPR and OFS provide information about material condition and structural response, respectively, bridge performance is also influenced by environmental and geotechnical processes extending beyond individual bridge assets. The following Section 6 therefore considers satellite remote sensing as a source of network-scale contextual information for multi-scale bridge monitoring.

## 6. Network-Scale Information Acquisition Using Satellite Remote Sensing

### 6.1. Satellite Remote Sensing as a Source of Network-Scale Context Information

Satellite remote sensing provides a means of observing bridge infrastructure and its surrounding environment over spatial and temporal scales that are difficult to achieve using ground-based monitoring alone. Within the framework established in Section 3, its principal contribution is the acquisition of network-scale contextual information, particularly information related to surface deformation, regional ground movement, environmental conditions, and infrastructure-wide spatial patterns.

Among satellite remote-sensing techniques, Synthetic Aperture Radar (SAR) interferometry has become particularly important for infrastructure monitoring because it can detect subtle surface deformation over large geographical areas. Interferometric SAR (InSAR) derives surface displacement information from the phase differences between radar observations acquired at different times. The development of Persistent Scatterer InSAR (PS-InSAR) and Small Baseline Subset (SBAS-InSAR) techniques has further improved the capability to monitor long-term deformation under complex observation conditions [108,109,110].

The principal advantage of satellite-based observation is its spatial coverage. Unlike local inspection and sensing systems, satellite observations can cover individual bridges together with their surrounding ground and infrastructure environment. Repeated acquisitions also provide a temporal dimension, allowing deformation histories and regional trends to be investigated over extended periods. Recent reviews have therefore highlighted the increasing contribution of remote sensing to bridge digitalisation and its integration with geomatics-based structural health monitoring frameworks [111,112].

**Figure 5:**
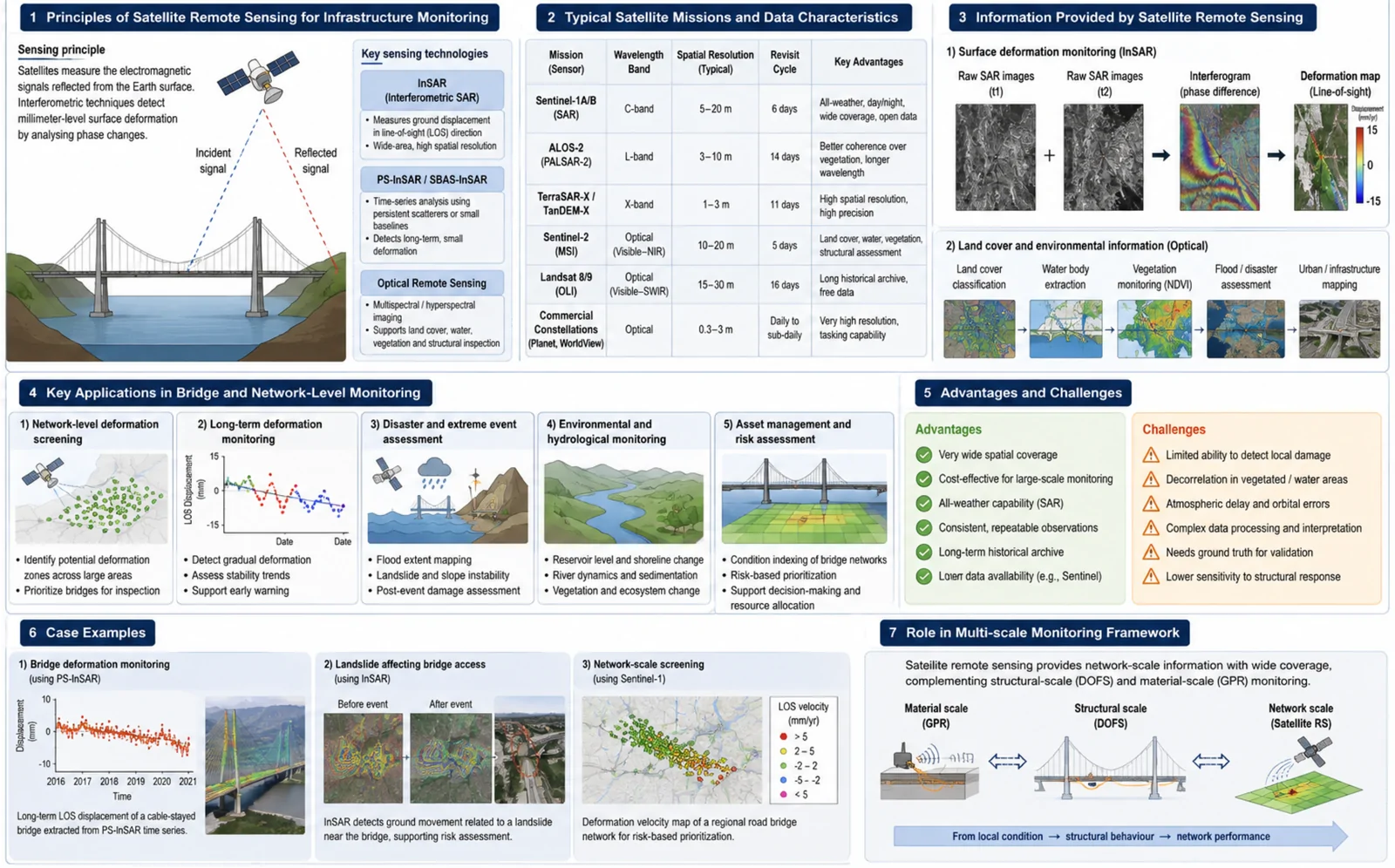
Satellite Remote Sensing for Regional-Scale Bridge Monitoring.

Different satellite observations provide complementary forms of information. Radar-based methods are particularly suitable for measuring surface deformation and displacement, whereas optical remote sensing can provide complementary information on surface characteristics and environmental conditions. In bridge applications, however, satellite-derived observations should not be interpreted as direct measurements of structural condition. InSAR measurements are primarily along the satellite line of sight (LOS), and their reliability can be affected by viewing geometry, coherent-scatterer availability, atmospheric effects, temporal and spatial decorrelation, reference-point stability, layover, and shadow. Figure 5 summarises the principal categories of Satellite Remote Sensing for bridge inspection and Digital Twin development.

These characteristics distinguish satellite remote sensing from material- and structural-scale sensing. Its primary value lies in providing a spatial and temporal context within which bridge behaviour can be evaluated. The following Section 6.2 therefore focuses on the principal categories of network-scale context information derived from satellite observations.

### 6.2. Network-Scale Context Information Acquired by Satellite Remote Sensing

Network-scale context information describes environmental, geographical, and infrastructure-wide processes that influence bridge performance beyond the boundaries of individual structural components. Satellite remote sensing is particularly suitable for acquiring such information because of its broad spatial coverage and repeated observations.

The information obtained from satellite observations can be organised into four complementary categories: surface deformation and displacement, long-term performance evolution, environmental and geohazard context, and network-level infrastructure information. These categories differ in their engineering interpretation but collectively provide a broader context for bridge assessment and infrastructure management.

#### 6.2.1. Surface Deformation and Displacement Information

Recent bridge-specific studies have demonstrated increasingly sophisticated InSAR applications for deformation monitoring. SBAS-InSAR has been applied to long-span railway bridges using Sentinel-1 observations, while PSI approaches have been refined using temperature fields and structural information to improve long-term deformation estimates [113,114]. Other studies have integrated structural knowledge with time-series InSAR to improve point density and distinguish mechanically meaningful deformation from environmental effects [115,116].

Surface deformation and displacement represent the most direct form of information obtained from satellite-based InSAR for bridge monitoring. Repeated observations can reveal spatially distributed deformation associated with bridge structures, foundations, surrounding ground, and nearby infrastructure.

The engineering value of satellite-derived displacement lies in its ability to provide observational evidence of long-term movement over areas that may be difficult to monitor comprehensively using ground-based systems. Progressive settlement, localised displacement, and regional deformation trends can be identified and subsequently evaluated using structural models, inspection records, and complementary monitoring observations.

Several studies have demonstrated the application of SBAS-InSAR to long-term deformation monitoring of bridges. Long-span railway bridges have been monitored using time-series InSAR, demonstrating the potential of satellite observations for tracking deformation under operational conditions [117]. InSAR-derived displacement measurements have also been used to support the assessment of urban bridges at the city scale [118]. At larger scales, regional InSAR monitoring can facilitate the screening of bridge inventories and the identification of structures exhibiting abnormal deformation behaviour [119].

However, satellite-derived displacement should not be interpreted directly as evidence of structural deterioration. The observed LOS displacement may include contributions from bridge deformation, foundation movement, surrounding ground deformation, thermal effects, and measurement uncertainty. Its engineering significance therefore depends on structural characteristics, geological conditions, environmental influences, and complementary observations.

Satellite-derived displacement should consequently be regarded as network-scale contextual evidence rather than a standalone diagnostic indicator of bridge damage.

#### 6.2.2. Long-Term Performance Evolution

Long-term bridge monitoring using satellite interferometry is particularly valuable because repeated observations can reveal deformation histories without installing permanent sensors on every asset. Multi-span prestressed concrete bridge studies have compared satellite-derived deformation with on-site measurements, while recent refined distributed-scatterer approaches have addressed low-coherence bridge groups and temperature-related phase effects [120,121]. These studies strengthen the case for treating satellite observations as network-scale contextual information rather than as isolated displacement measurements.

An important characteristic of satellite remote sensing is the ability to establish deformation histories from repeated observations. Whereas conventional inspection campaigns often provide discrete assessments of bridge condition, time-series satellite observations can reveal how deformation evolves over months or years.

Long-term displacement trajectories provide information that is difficult to obtain from individual observations. They can help distinguish relatively stable behaviour from progressive deformation, seasonal variations, or abnormal trends requiring further investigation. This temporal perspective is particularly valuable for infrastructure management because slowly developing changes may remain difficult to identify through isolated inspection campaigns.

The interpretation of long-term performance evolution nevertheless requires careful consideration of environmental and operational influences. Seasonal thermal effects, foundation movement, regional geological processes, and changes in surrounding conditions may all contribute to observed displacement histories. Long-term trends should therefore be interpreted together with structural characteristics, environmental conditions, operational loading, maintenance records, and complementary monitoring observations.

Within the network-scale context provided by satellite remote sensing, temporal deformation histories add an important longitudinal dimension to bridge assessment. They support trend analysis, performance evaluation, and the identification of structures requiring closer investigation.

#### 6.2.3. Environmental and Geohazard Context

Environmental interpretation is increasingly recognised as essential for InSAR-based bridge monitoring. Temperature, ground movement, riverbed processes, and surrounding geohazards can influence observed bridge displacement and therefore need to be separated from structural deterioration. Regional-scale studies have explicitly combined InSAR displacement with environmental variables to classify anomalous bridge behaviour, while broader reviews have identified climate and environmental context as important components of regional-scale bridge health monitoring [19,122].

Bridge performance is influenced by the environmental and geotechnical conditions surrounding individual assets. Ground settlement, slope instability, flooding, river morphology, seismic effects, and other regional processes may alter bridge behaviour without originating from deterioration within the bridge itself.

Satellite remote sensing provides a means of observing such processes over large geographical areas. The resulting information can help establish relationships between bridge behaviour and its surrounding environment. For example, interferometric SAR has been investigated for remote monitoring of bridge-scour-related hazards, demonstrating the potential of satellite observations for identifying environmental conditions that are difficult to evaluate through conventional inspection alone [123].

The engineering value of environmental and geohazard information lies primarily in its contextual role. Regional ground movement may explain an observed bridge displacement, while spatially extensive environmental changes may distinguish a local structural problem from a broader geotechnical process. Such information can therefore improve the interpretation of bridge-level observations and support risk assessment.

Satellite observations should nevertheless be interpreted together with appropriate environmental, geological, and structural information. The presence of an environmental anomaly does not by itself establish a structural consequence, and its significance depends on the vulnerability and configuration of the affected bridge.

#### 6.2.4. Network-Level Infrastructure Assessment and Inspection Prioritisation

The network-scale potential of satellite monitoring is particularly evident in population-based approaches. Regional studies have investigated anomaly detection across multiple bridges, while recent work has extended the concept to historical bridge populations and large-area health assessment [19,122,124]. Such approaches can provide an initial prioritisation layer, identifying assets that require more detailed structural monitoring or material-scale inspection.

The broad spatial coverage of satellite remote sensing enables multiple bridges to be evaluated within a common geographical and temporal framework. This capability extends bridge assessment from individual assets towards network-level screening and infrastructure management.

Network-scale observations can reveal regional deformation patterns and differences in the behaviour of bridges subjected to similar environmental and operational conditions. MT-InSAR observations combined with machine-learning techniques have been investigated for multi-bridge deformation monitoring and for identifying bridges exhibiting abnormal behavioural characteristics [125]. Such comparative analysis can reduce the dependence on independent assessment of every individual asset and facilitate the identification of structures requiring further investigation.

The principal engineering value of network-level assessment is therefore comparative rather than diagnostic. Bridges exhibiting deformation characteristics that differ substantially from neighbouring assets or regional trends can be prioritised for detailed inspection. This can assist transportation agencies in allocating inspection resources, scheduling maintenance, and identifying infrastructure requiring closer engineering evaluation.

Satellite remote sensing is consequently better viewed as a network-level screening and contextualisation technology than as a replacement for detailed bridge inspection. Its contribution is to identify spatial and temporal patterns that can guide subsequent engineering investigation and asset-management decisions.

### 6.3. Intelligent Interpretation of Network-Scale Information

Intelligent InSAR interpretation is also moving towards data fusion and three-dimensional reconstruction. Recent work has combined multi-track InSAR observations with environmental and structural information through Kalman filtering, while other studies have used deep learning and attention mechanisms to derive bridge-failure risk indicators from geospatial displacement time series [126,127]. These developments indicate that satellite observations can become higher-level engineering information when combined with structural knowledge and complementary data sources.

The increasing availability of satellite observations has shifted an important part of the monitoring challenge from data acquisition towards information interpretation. Large Earth-observation datasets contain extensive spatial and temporal information, but converting these observations into engineering evidence requires appropriate pattern recognition, contextual analysis, and uncertainty assessment.

Machine-learning techniques have increasingly been applied to network-scale bridge monitoring. MT-InSAR observations can be analysed using supervised or unsupervised approaches to identify abnormal deformation patterns and organise bridges according to their observed behaviour. For example, unsupervised clustering has been investigated for grouping bridge-deformation patterns and screening large bridge inventories for structures requiring further investigation [128].

The main advantage of intelligent interpretation is therefore its ability to process large numbers of observations consistently. Rather than analysing each deformation trajectory independently, data-driven methods can identify common patterns, outliers, and spatial relationships across bridge inventories.

However, deformation anomalies cannot be interpreted as engineering conclusions without additional context. Similar displacement patterns may result from structural deterioration, foundation settlement, environmental loading, nearby construction, or measurement uncertainty. Conversely, the same deterioration mechanism may produce different satellite signatures under different structural and environmental conditions.

Reliable intelligent interpretation therefore requires the integration of satellite observations with complementary engineering information. Historical inspection records provide evidence of previous condition and maintenance, bridge databases describe structural characteristics and operational conditions, environmental observations establish regional context, and numerical models provide physical constraints. Combining satellite observations with terrestrial monitoring can improve engineering assessment compared with relying on a single information source [129]. Bayesian data-fusion approaches further provide a means of integrating multiple observations while explicitly accounting for uncertainty in deformation estimation and interpretation [130].

From an information-centric perspective, AI should therefore serve primarily as an interpretation and information-organisation tool. Its role is to identify meaningful patterns, quantify uncertainty, and provide evidence that can support engineering judgement rather than to replace that judgement.

### 6.4. Current Challenges

Despite significant advances in satellite remote sensing, several challenges remain before network-scale observations can be routinely converted into reliable engineering information for bridge management.

First, the relationship between observed surface deformation and actual structural condition remains non-unique. Satellite observations quantify surface movement but do not directly identify the physical mechanism responsible for that movement. Internal deterioration may produce little observable surface displacement, while similar deformation patterns may result from structural, geotechnical, or environmental processes.

Second, the interpretation of long-term deformation is affected by multiple coupled factors. Seasonal thermal effects, atmospheric disturbances, foundation movement, regional geological processes, and structural response may all contribute to the measured signal. Separating these effects remains essential for reliable engineering interpretation.

Third, the reliability and coverage of satellite observations depend on observation geometry and surface conditions. LOS measurements do not directly provide complete three-dimensional displacement, while the availability of coherent targets can be limited for some bridge components. Atmospheric effects, decorrelation, layover, shadow, and reference-point selection can further affect the quality of deformation estimates.

Fourth, network-scale monitoring generates large volumes of spatiotemporal information that require efficient storage, processing, and interpretation. Although machine learning can support large-scale screening, models must remain transferable across different bridge types, geographical environments, observation geometries, and deformation mechanisms.

Finally, satellite observations cannot provide a complete representation of bridge condition independently. Their greatest value is obtained when network-scale context is combined with material-condition information, structural-response observations, environmental data, and engineering models. Establishing reliable interfaces between these information sources remains an important challenge for multi-scale bridge monitoring.

### 6.5. Role of Satellite Remote Sensing in the Multi-Scale Bridge Monitoring Framework

The recent literature therefore supports a distinct role for satellite remote sensing within the M–S–N framework. Satellite InSAR is not a replacement for local material inspection or structural instrumentation; rather, it provides wide-area temporal and environmental context that can identify unusual behaviour, support cross-bridge comparison, and prioritise subsequent investigation [15,19,122,124,126,131,132]. Recent reviews of bridge digitalisation likewise emphasise the value of combining remote sensing with BIM, Digital Twins, and other monitoring technologies [15].

Within the multi-scale framework established in Section 3, satellite remote sensing provides network-scale contextual information that extends bridge monitoring beyond individual structural components. Its principal contribution is the observation of deformation, environmental conditions, and infrastructure-wide spatial and temporal patterns that cannot be efficiently captured by local sensing systems.

The information obtained from satellite observations is complementary to the material-condition information provided by GPR and the structural-response information obtained from OFS. Satellite remote sensing does not directly replace either technology. Instead, it provides the geographical and environmental context required to interpret local material conditions and structural responses within a broader infrastructure system.

The progression from individual deformation measurements to time-series analysis and network-level screening also reflects the information-centric evolution of bridge monitoring. Satellite observations can first provide measurable deformation evidence, which can then be interpreted in relation to environmental and structural context and ultimately used to support inspection prioritisation and infrastructure management.

The principal role of satellite remote sensing in the proposed framework is therefore to provide network-scale contextual evidence. Its value is maximised when this information is linked with material-condition and structural-response observations through a common information framework. The integration of these heterogeneous information sources is considered in the following Section 7.

## 7. Comparison Across Different Spatial Scales

### 7.1. Monitoring Objectives Across Multiple Spatial Scales

Bridge-monitoring technologies differ not only in sensing principles but also in the type of engineering information they can provide, their spatial coverage, and their temporal characteristics. The comparison in this Section 7 therefore focuses on the information obtained at different spatial scales rather than on sensor specifications alone.

Table 3 summarises the principal monitoring objectives associated with GPR, optical-fibre sensing, and satellite remote sensing. The comparison shows that the three technologies address different but complementary information requirements. GPR primarily supports material-condition assessment, OFS provides structural-response information, and satellite remote sensing contributes network-scale contextual information. In some applications, a monitoring objective may involve more than one information scale.

The overlap between information categories is important because bridge deterioration and structural behaviour are not independent processes. Material deterioration may eventually alter structural response, while structural deformation may be influenced by regional ground movement or environmental processes. Consequently, some engineering questions cannot be adequately addressed at a single spatial scale. For example, crack-related assessment may require both information on local material deterioration and measurements of the associated structural response, whereas deformation assessment may benefit from combining structural observations with network-scale ground-movement information [90,96,125].

The comparison therefore indicates that the three sensing approaches should not be regarded as competing alternatives. Their engineering value depends on the relationship between the monitoring objective and the information required to address it.

### 7.2. Practical Considerations

In addition to the type of engineering information obtained, the three sensing approaches differ substantially in spatial coverage, spatial resolution, temporal sampling, and deployment requirements. These characteristics determine where and when each technology can be effectively applied.

GPR is primarily a campaign-based, component-level technique. It provides relatively detailed information along surveyed trajectories and is particularly suitable for targeted investigation of bridge components where hidden deterioration or material conditions require assessment. Its main limitation is that repeated surveys require physical access and are generally performed periodically rather than continuously.

OFS provides localised or distributed structural measurements, depending on the sensing configuration. Permanently installed fibre systems can support continuous monitoring over extended periods, making them particularly suitable for tracking structural responses under operational and environmental loading. However, deployment generally requires physical installation on or within the monitored structure, and the monitoring coverage is determined by the location and configuration of the sensing system.

Satellite remote sensing provides large-area, remote observations that can cover individual bridges together with surrounding infrastructure and ground conditions. Repeated satellite acquisitions enable long-term deformation histories and network-level comparisons without requiring direct physical access to the monitored assets. Its spatial and temporal resolution, however, is constrained by satellite acquisition characteristics, observation geometry, coherent-scatterer availability, and environmental conditions. These differences are summarised in Table 4.

The differences in deployment and observation characteristics imply that technology selection should be driven by the monitoring objective rather than by sensing capability alone. GPR is advantageous when detailed material-condition information is required; OFS is more appropriate when continuous structural response is the primary target; and satellite remote sensing is particularly valuable when broad spatial coverage and long-term contextual information are required.

### 7.3. Information Characteristics Across Scales

The comparison also reveals that the three technologies have different information strengths and limitations. No individual sensing approach provides a complete representation of bridge condition because each observes only part of the physical system.

GPR can provide information on subsurface and material-level conditions that may not yet produce a measurable change in global structural response. However, the presence of a detected defect does not necessarily indicate its immediate influence on structural performance. OFS provides direct observations of structural response under operational and environmental loading, but abnormal response alone may not uniquely identify the underlying deterioration mechanism. Satellite remote sensing extends observations to the surrounding ground and infrastructure network, but observed deformation cannot generally be attributed to a specific structural cause without additional engineering information.

These differences create information gaps rather than simple sensor limitations. A material anomaly identified by GPR may require structural-response measurements to determine its significance. An abnormal OFS response may require material inspection or network-scale observations to distinguish structural deterioration from environmental or geotechnical effects. Similarly, satellite-derived deformation may require structural and material information to determine whether the observed movement originates from the bridge, its foundation, or the surrounding ground.

Accordingly, the complementarity of the three approaches can be summarised as follows: GPR identifies what may be deteriorating; OFS characterises how the structure responds; satellite remote sensing provides the broader spatial and environmental context in which that response occurs.

This complementarity is the principal justification for multi-scale monitoring. The objective is not to maximise the number of sensing technologies, but to reduce critical information gaps by combining observations that describe different aspects of the same infrastructure system.

### 7.4. Comparative Discussion

The practical selection of monitoring technologies depends on the engineering question, required information, spatial extent, monitoring duration, accessibility, and operational constraints. The suitability of each technology depends on the bridge type, deterioration mechanism, environmental conditions, sensing configuration, and required information. In practice, the most effective monitoring strategy may combine technologies with different strengths rather than relying on a single modality.

For example, satellite observations can first be used to identify bridges or surrounding areas exhibiting abnormal deformation or environmental changes. OFS can then provide continuous structural-response measurements for selected assets, while GPR can be deployed for targeted investigation of material deterioration. Such a staged strategy links network-level screening with structural monitoring and detailed material assessment, potentially improving the efficiency of inspection and maintenance resources.

### 7.5. Implications for Multi-Source Integration

The expanded literature reveals an increasing number of studies that combine heterogeneous observations, although most remain focused on particular sensor pairs or individual monitoring objectives. For example, InSAR has been combined with terrestrial measurements and topographic observations, while recent bridge-deck research has demonstrated GPR–IRT fusion for delamination detection [57,132,133]. These studies provide practical evidence that complementary measurements can reduce the ambiguity inherent in single-sensor interpretation, but they do not yet establish a general framework linking material, structural, and network-scale information.

The comparison across spatial scales leads to an important distinction between measurement capability and information value. The three technologies differ substantially in what they measure, how they observe it, and the spatial and temporal scales at which observations are available. Their outputs therefore cannot simply be merged as equivalent data streams.

Instead, each observation should retain its physical meaning, spatial reference, temporal characteristics, and associated uncertainty. GPR observations may describe a local material condition, OFS measurements may represent structural response at a specific location and time, while satellite observations may describe regional deformation or environmental context. Their integration therefore requires a framework capable of preserving these differences while establishing relationships between them.

This comparison provides the basis for the multi-source integration framework presented in the following Section 8. Rather than treating GPR, OFS, and satellite remote sensing as independent monitoring systems, the integration framework considers their outputs as heterogeneous but complementary sources of engineering evidence. The objective is to transform these observations into a consistent representation that can support condition assessment, structural-state interpretation, and Digital Twin updating.

## 8. Multi-Source Integration Framework for Intelligent Bridge Monitoring

The comparison in Section 7 demonstrates that GPR, optical-fibre sensing, and satellite remote sensing provide complementary engineering information at different spatial and temporal scales. The central challenge is therefore not simply to acquire additional measurements, but to establish meaningful relationships among heterogeneous observations and transform them into a consistent representation of bridge condition and performance. This requires integration at the levels of spatial and semantic registration, temporal alignment, information fusion, intelligent interpretation, and Digital Twin updating.

### 8.1. Multi-Scale Integration Framework

**Figure 6:**
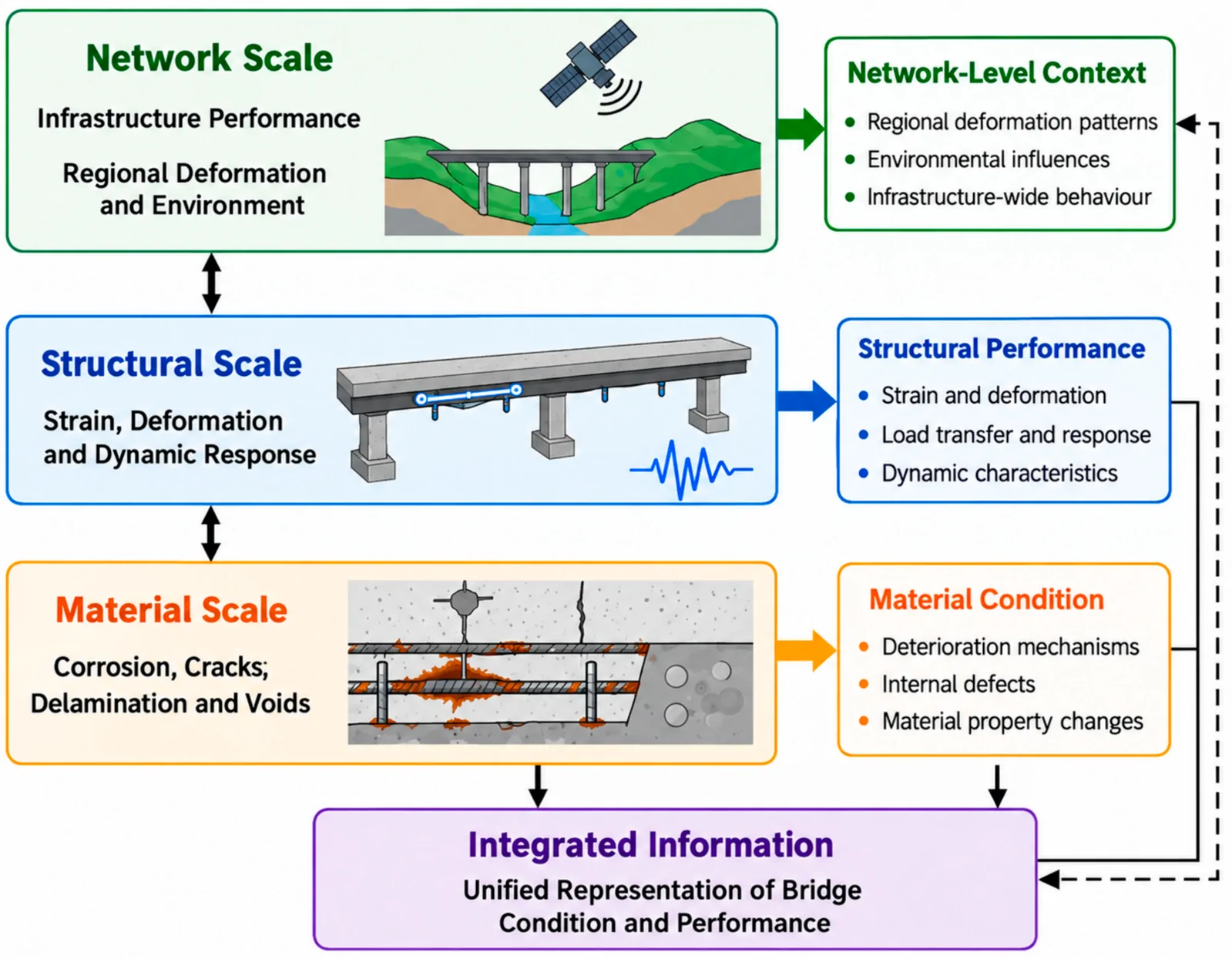
Multi-scale integration framework for bridge monitoring.

The proposed framework organises bridge-monitoring information into three interconnected layers: material-condition information, structural-response information, and network-scale context information. Rather than treating these layers as independent monitoring domains, the framework considers them as complementary descriptions of the same physical infrastructure system. Building upon these developments, this review proposes the multi-scale integration framework illustrated in Figure 6.

At the material scale, observations describe deterioration mechanisms, internal defects, moisture conditions, and changes in material properties. At the structural scale, monitoring observations characterise strain, deformation, temperature, load transfer, and dynamic response. At the network scale, observations provide broader geographical and environmental context, including regional deformation, ground movement, and infrastructure-wide trends. The relationships between these layers are bidirectional in the sense that local deterioration may influence structural response, while environmental and geotechnical processes at larger scales may affect the behaviour of individual bridges.

For multi-source integration, heterogeneous observations first need to be associated with a common spatial and semantic reference system. GPR measurements can be referenced to surveyed inspection trajectories and bridge components, fibre measurements can be associated with their physical installation locations and structural elements, and satellite observations can be georeferenced to corresponding bridge or surrounding-ground locations where the observation characteristics permit. Each observation should additionally retain essential metadata, including sensing modality, measurement quantity, acquisition time, spatial location, structural component, and uncertainty.

This organisation allows the integration process to operate on engineering information rather than raw sensor measurements alone. Measurements are first converted into interpretable information, such as material-condition indicators, structural-response parameters, or deformation trends, and are then associated with the corresponding components and states of the bridge Digital Twin. The resulting representation provides a common basis for subsequent data fusion and intelligent interpretation.

### 8.2. Multi-Source Data Fusion

Recent work provides stronger empirical support for multi-source fusion in bridge monitoring. Combined InSAR and terrestrial monitoring has demonstrated how satellite observations can be directly compared with in situ measurements, while Bayesian fusion has been used to combine InSAR and topographic displacement measurements [132,133]. At the material scale, GPR and infrared thermography have recently been integrated through cross-modal attention and physics-enhanced fusion for bridge-deck delamination detection [57]. These examples illustrate three complementary fusion requirements: spatial registration, temporal consistency, and engineering-level interpretation of measurements with different uncertainty characteristics.

The three sensing modalities differ substantially in spatial resolution, temporal sampling, measurement uncertainty, and data format. Directly combining their raw measurements is therefore generally inappropriate. Instead, fusion should preserve the physical meaning and acquisition characteristics of each information source while establishing relationships between observations that describe related infrastructure states.

Spatial fusion establishes relationships between observations acquired at different scales. Material-condition information can be associated with specific bridge components, structural-response observations can be linked to the corresponding structural elements, and network-scale observations can provide environmental and geotechnical context. Such spatial relationships allow local deterioration and structural behaviour to be interpreted together with broader infrastructure conditions.

Temporal fusion is equally important because the three technologies operate at different monitoring frequencies. Continuous structural measurements from OFS may provide high-frequency or long-duration observations, whereas GPR surveys and satellite observations are generally episodic. Rather than forcing these datasets onto a common sampling interval, the integration framework should maintain a time-indexed representation in which each observation retains its acquisition time, observation interval, and data latency. High-frequency structural measurements can be converted into event-level or statistical descriptors when appropriate, while periodic GPR and satellite observations can be incorporated as condition or deformation updates.

Physics-based fusion provides an additional layer of integration by imposing engineering relationships between the different observations. Structural mechanics, electromagnetic propagation, environmental processes, and numerical models can provide constraints for interpreting heterogeneous observations. For example, a material-condition anomaly identified by GPR may be evaluated in relation to observed structural response, while satellite-derived ground deformation may provide context for interpreting changes in bridge response. Such relationships can improve physical consistency and reduce the risk of treating correlated observations as independent evidence.

Uncertainty should be retained throughout the fusion process. Measurements from different sensing systems have different error characteristics, coverage limitations, and reliability. Sensor-specific uncertainty can therefore be represented through measurement variance, data-quality indicators, confidence scores, or context-dependent weighting. Missing or unreliable observations should be identified explicitly rather than indiscriminately replaced. Where appropriate, interpolation, spatial inference, or model-based prediction can be applied while maintaining the associated uncertainty.

The objective of multi-source fusion is consequently not to maximise the amount of monitoring data, but to establish a more consistent and physically meaningful representation of bridge condition and performance. This provides the information foundation for intelligent interpretation and Digital Twin updating.

### 8.3. AI-Enabled Intelligent Interpretation

AI is increasingly being used not only for single-sensor classification but also for feature fusion and cross-modal interpretation. Recent bridge studies have employed multi-domain feature fusion, unsupervised deep learning, and multimodal learning to identify subtle anomalies and improve generalisation across structures [14,29,57]. The implication for the M–S–N framework is that AI should operate on engineering information derived from multiple sensing scales, rather than treating heterogeneous raw observations as interchangeable inputs.

Multi-source integration does not by itself produce engineering knowledge. Heterogeneous observations still need to be interpreted in relation to deterioration mechanisms, structural behaviour, environmental conditions, and operational requirements. Within the proposed framework, AI therefore functions as an interpretation layer between information fusion and engineering decision support.

Multimodal learning provides one possible approach for learning relationships between material-condition information, structural-response measurements, network-scale observations, inspection records, and numerical simulations. Such models may identify patterns that are difficult to capture through independent analysis of each sensing modality and may improve robustness when individual information sources are incomplete.

Physics-informed intelligence provides a complementary strategy by incorporating engineering knowledge into data-driven models. Structural mechanics, electromagnetic principles, numerical simulation, and engineering constraints can be used to restrict model behaviour and improve interpretability. This is particularly important for bridge monitoring because correlations identified from monitoring data do not necessarily correspond to physical deterioration mechanisms.

As heterogeneous infrastructure datasets continue to expand, more general multimodal and transferable learning approaches may become relevant. Foundation models and related representation-learning approaches offer the potential to learn common representations from diverse monitoring data and transfer knowledge across bridge types, sensing systems, and monitoring scenarios. However, their practical application to bridge infrastructure remains subject to challenges involving data availability, domain transfer, physical consistency, uncertainty, and interpretability.

AI should therefore be regarded as an analytical assistant rather than an autonomous decision-maker. Its principal role is to identify patterns, estimate latent infrastructure states, quantify uncertainty, and provide interpretable evidence for engineering assessment. In this framework, AI connects multi-source information fusion with Digital Twin updating and lifecycle decision support.

### 8.4. Digital Twin Updating and Decision Support

New Digital Twin implementations further support this direction. Data-driven bridge Digital Twins have been coupled with unsupervised anomaly detection, multi-sensor fusion, and edge–cloud architectures, while recent bridge-management research has linked Digital Twins and deep learning to maintenance planning and uncertainty management [14,25,29]. These developments provide an emerging technical basis for the proposed information pathway from sensing to information, from information to Digital Twin state updating, and from updated state estimates to maintenance decision support.

The integrated information generated through multi-source monitoring ultimately needs to be incorporated into the bridge Digital Twin. Digital Twin updating provides the mechanism through which observations from the physical bridge modify the digital representation and maintain consistency between the two.

Updating may occur at different levels. Monitoring observations can first update condition indicators and state variables, followed by the recalibration of numerical or data-driven models where sufficient evidence is available. Historical inspection records, maintenance activities, environmental conditions, and operational information can be incorporated alongside sensor observations to provide a more complete representation of the current bridge state.

Measurement-informed model updating can reduce discrepancies between predicted and observed structural behaviour. However, updating should not be interpreted as an automatic replacement of model parameters whenever new measurements become available. The reliability of an update depends on data quality, parameter identifiability, model uncertainty, and the consistency between the observed response and the assumed physical model. Verification and uncertainty assessment are therefore important components of the updating process.

**Figure 7:**
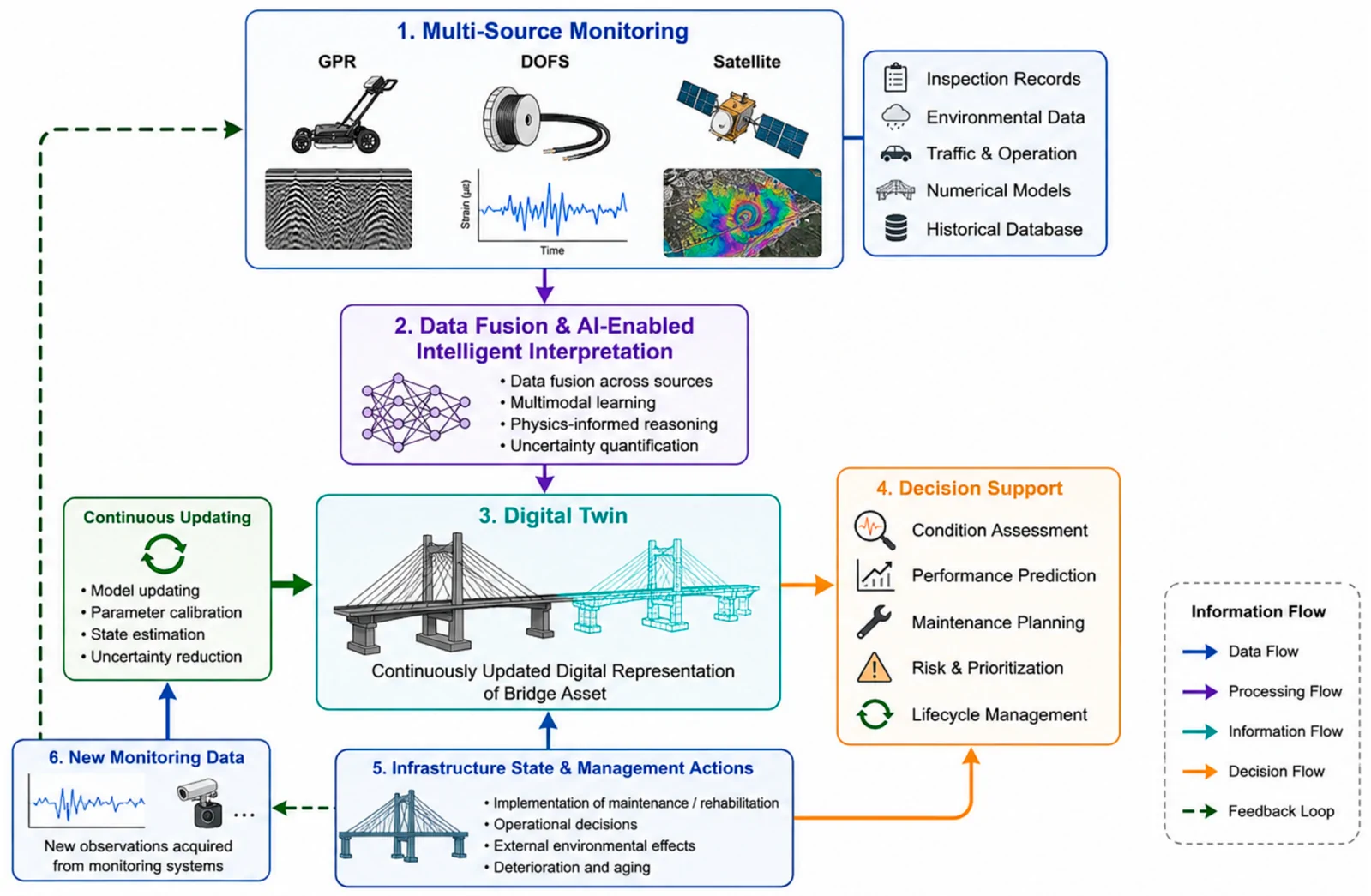
Self-evolving Digital Twin framework for intelligent bridge management.

Once updated, the Digital Twin can support several engineering functions, including condition assessment, performance prediction, inspection prioritisation, maintenance planning, and risk-informed decision support. The resulting process can be represented as a closed information cycle: monitoring observations, information extraction, multi-source fusion, intelligent interpretation, Digital Twin updating, engineering decision support, new observations. The self-evolving Digital Twin concept illustrated in Figure 7 summarises this process.

This closed-loop structure distinguishes an information-centric bridge Digital Twin from a static digital representation. The Digital Twin is not simply a repository for monitoring data; it provides a structured environment in which heterogeneous observations can be interpreted, models can be updated, and engineering decisions can be informed by continuously evolving evidence.

The framework therefore shifts the focus of bridge monitoring from sensor deployment towards knowledge generation. The objective is not to replace individual monitoring technologies, but to establish a coherent information pathway through which complementary observations can contribute to a continuously updated representation of bridge condition, structural performance, and infrastructure context.

## 9. Future Perspectives

The framework presented in Section 8 establishes an information-centric approach for integrating heterogeneous bridge-monitoring observations. Future developments are expected to extend this framework beyond coordinated sensing and information fusion towards increasingly autonomous, connected, and continuously evolving monitoring systems. The main directions include autonomous information acquisition, connected monitoring ecosystems, adaptive monitoring, and self-evolving bridge Digital Twins.

### 9.1. From Manual Inspection to Autonomous Information Acquisition

Bridge inspection has traditionally relied on periodic field surveys performed by engineers and inspectors. Although manual inspection remains essential for engineering evaluation, it can be labour-intensive, time-consuming, and constrained by accessibility, operational safety, and the availability of skilled personnel. Advances in autonomous sensing platforms are creating opportunities to supplement conventional inspection with more frequent and flexible information acquisition.

Uncrewed Aerial Systems (UASs) have become important platforms for inspecting difficult-to-access bridge components and can reduce exposure of inspectors to hazardous environments. Autonomous ground robots and climbing robots provide additional access to bridge decks, girders, piers, and other structural components. These platforms can carry cameras, LiDAR, GPR, and other sensing systems, enabling multiple forms of information to be acquired within a single inspection mission [134,135].

Autonomous inspection is particularly relevant to sensing technologies that traditionally require substantial manual intervention. Mobile robotic platforms equipped with GPR and other nondestructive testing technologies have demonstrated the feasibility of combining autonomous mobility with multi-sensor perception [136,137]. Such systems can improve measurement repeatability and enable observations to be collected in locations or under conditions that are difficult to access through conventional inspection.

The development of autonomous inspection is also increasingly connected to multi-sensor information processing. Recent reviews and robotic inspection studies emphasise the integration of mobile sensing, AI, and infrastructure Digital Twins, suggesting that future autonomous platforms could use network-scale screening to select assets, structural-response monitoring to identify abnormal behaviour, and targeted GPR or other NDT measurements to investigate material-scale causes [27,29,104]. This hierarchical strategy is consistent with the M–S–N framework and provides a practical route from wide-area screening to targeted inspection.

The longer-term objective is not simply to automate existing inspection procedures, but to develop intelligent information-acquisition systems that can determine where and when additional observations are required. This would allow routine monitoring to identify areas of concern and subsequently guide targeted robotic or mobile inspections. Autonomous information acquisition could therefore complement, rather than replace, engineering inspection by improving the availability and consistency of evidence for bridge assessment.

### 9.2. From Isolated Monitoring Systems to Connected Monitoring Ecosystems

Current bridge-monitoring systems are often developed around individual technologies or specific inspection objectives. Future systems are likely to become more interconnected, allowing permanently installed sensors, mobile inspection platforms, satellite observations, inspection records, maintenance databases, and engineering models to exchange information through shared digital infrastructures.

A connected monitoring ecosystem would enable information acquired at different spatial and temporal scales to be coordinated within a common framework. For example, network-scale observations could identify bridges requiring closer investigation, structural monitoring could then provide continuous response information, and targeted GPR or robotic inspection could subsequently investigate specific material conditions. The results of these investigations could be returned to the shared information environment and used to update monitoring priorities.

Such connectivity requires more than communication between sensors. Interoperability of data structures, semantic consistency, spatial registration, temporal synchronisation, uncertainty representation, and access to engineering models are all necessary for reliable information exchange. The development of interoperable digital infrastructures is therefore likely to become increasingly important as bridge monitoring moves from independent sensing systems towards coordinated infrastructure-information ecosystems.

The ultimate objective is to establish a monitoring environment in which inspection, sensing, analysis, and asset management are connected through continuous information exchange. This would provide the infrastructure required for more responsive bridge management at both individual-asset and transportation-network scales.

### 9.3. Towards Autonomous Monitoring Systems

Connected monitoring ecosystems provide the foundation for a further transition towards adaptive monitoring. Rather than following fixed inspection schedules, future monitoring systems may adjust their sensing activities according to bridge condition, environmental conditions, operational demands, and previously acquired observations.

Edge intelligence is one enabling technology for this transition. Processing monitoring data close to the point of acquisition can support rapid anomaly detection, data filtering, and preliminary condition assessment while reducing communication requirements and latency. Lightweight SHM systems based on edge computing have already demonstrated the potential of distributed processing for infrastructure monitoring [138].

Autonomous sensing platforms can further extend this capability. UASs, robotic monitoring systems, and mobile NDT platforms can be deployed when additional information is required [134,135,136,137,138,139]. For example, a persistent monitoring system could identify an abnormal structural response and automatically generate an inspection request for a mobile platform. The newly acquired observations could then be used to reassess the condition and determine whether further monitoring is necessary.

This creates an adaptive monitoring loop: continuous observation, anomaly identification, targeted inspection, information update, monitoring reconfiguration.

Such a system could improve the allocation of inspection resources by directing detailed measurements towards locations where additional information is most valuable. However, increasing automation should not imply the removal of human engineering responsibility. Safety assessment, uncertainty evaluation, interpretation of ambiguous evidence, and decisions with significant operational consequences require appropriate engineering oversight.

Future bridge monitoring is therefore more likely to follow a human–AI–robot collaborative paradigm, in which autonomous systems perform routine acquisition and analysis while engineers retain responsibility for validation, interpretation, and strategic decisions.

### 9.4. Towards Self-Evolving Digital Twins

The long-term development of the framework is the emergence of bridge Digital Twins that can continuously adapt their representation and engineering knowledge as new evidence becomes available. This concept extends beyond conventional model updating. A self-evolving Digital Twin would progressively incorporate monitoring observations, inspection records, environmental conditions, operational information, maintenance history, and engineering models to refine its representation of bridge condition and performance.

A key requirement is continuous knowledge assimilation. As new observations are acquired, the Digital Twin should be able to determine whether they confirm existing assumptions, indicate a change in infrastructure state, or reveal inconsistencies requiring further investigation. This requires mechanisms for uncertainty-aware data assimilation, model validation, parameter updating, and conflict resolution between heterogeneous information sources.

AI may provide important capabilities for this evolution. Physics-informed learning, uncertainty-aware inference, explainable AI, and continual learning can support the extraction of new relationships from accumulated infrastructure data while maintaining physical consistency and engineering interpretability. Edge and cloud computing can provide complementary computational resources for real-time processing and more computationally demanding model updating.

Recent physics-informed SHM research further suggests that future Digital Twins will need to combine data-driven learning with engineering constraints, uncertainty quantification, model validation, and continuous updating [26]. This is particularly important for a multi-scale system because information from GPR, optical-fibre sensing, and satellite observations has different error structures, spatial resolutions, and physical meanings. A self-evolving Digital Twin should therefore update engineering states through traceable data assimilation rather than direct unconstrained modification of model parameters.

The concept of self-evolution should nevertheless be interpreted carefully. A continuously updated Digital Twin does not imply unrestricted autonomous modification of engineering models or management decisions. Changes to critical model parameters, structural-state estimates, or safety-related conclusions should remain subject to appropriate validation and engineering oversight. The reliability of a self-evolving system therefore depends on transparent update mechanisms, traceable data provenance, uncertainty quantification, and clearly defined human intervention points.

At the network level, individual bridge Digital Twins may eventually become interconnected with transportation, environmental, and infrastructure-management systems. Information exchanged among multiple assets could support corridor-level performance assessment, resilience analysis, coordinated maintenance planning, and network-level risk management.

The long-term evolution can therefore be viewed as a progression from data acquisition to information integration, from information integration to engineering knowledge, and from engineering knowledge to adaptive decision support. In this context, self-evolving Digital Twins represent a long-term vision in which sensing technologies, engineering models, AI, autonomous systems, and human expertise operate as components of a continuously learning infrastructure-management ecosystem.

## Figures and Tables

**Table 1:** Scale–information–technology mapping framework for bridge Digital Twins.

Engineering Information	Spatial Scale	Typical Parameters	Representative Technology	Digital Twin Function
Material-condition information	Material scale	Corrosion, delamination, voids, moisture	GPR	Condition assessment
Structural-response information	Structural scale	Strain, deflection, vibration, cable force	DOFS	Structural state updating
Network-scale contextual information	Network scale	Ground settlement, regional deformation, environmental hazards	Satellite remote sensing	Environmental context awareness

**Table 2:** Unified classification of material-condition information derived from GPR.

Information Category	Representative Engineering Target	Physical Interpretation	Representative GPR Signature	Conventional Interpretation Strategy	Emerging Research Direction
Geometry Information	Reinforcement localisation	Structural geometry	Hyperbolic reflections	Hyperbola fitting and cover estimation	Automatic geometric extraction
Material Deterioration Information	Corrosion assessment	Material degradation	Reflection attenuation, waveform variation	Statistical and amplitude-based analysis	Quantitative deterioration evaluation
Interface Integrity Information	Delamination and debonding	Layer continuity	Interface reflections, lateral-wave effects	Visual interpretation and migration	Physics-informed characterisation
Internal Cavity Information	Void detection	Hidden cavities and support conditions	Strong dielectric contrast and anomalous reflections	Reflection analysis and imaging	Multi-parameter characterisation
Material Property Information	Moisture assessment	Dielectric-property distribution	Travel-time variation and signal attenuation	Dielectric estimation	Quantitative material-property inversion

**Table 3:** Comparative monitoring objectives of multi-scale bridge sensing technologies.

Information Category	Representative Engineering Objective	Primary Information Scale	Typical Technology
Material-condition information	Corrosion, delamination, voids	Material	GPR
Material-condition + Structural-response information	Crack evolution	Material–Structural	GPR + DOFS
Structural-response information	Strain, prestress, load transfer	Structural	DOFS
Structural-response + Network-scale context information	Deformation assessment	Structural–Network	DOFS + Satellite
Network-scale context information	Settlement, regional deformation	Network	Satellite

**Table 4:** Practical characteristics of multi-scale bridge sensing technologies.

Characteristic	GPR	DOFS	Satellite
Physical access	Required	Required (installation)	Not required
Monitoring mode	Periodic	Continuous	Periodic revisit
Typical coverage	Component	Bridge	Network
Scalability	Moderate	Moderate	High
Long-term monitoring	Limited	Excellent	Good
Typical deployment	Campaign-based	Permanent installation	Remote observation

## Data Availability

No new data were created or analyzed in this study. Data sharing is not applicable to this article.

## Abbreviations

The following abbreviations are used in this manuscript

GPR	Ground-Penetrating Radar
DOFS	Distributed optical-fibre sensing
M-S-N	Material–Structural–Network
SHM	Structural health monitoring
NDT	Nondestructive testing
PS-InSAR	Persistent Scatterer Interferometric Synthetic Aperture Radar
CMP	Common Mid-Point
AI	Artificial intelligence
PINNs	Physics-Informed Neural Networks
FDTD	Finite-difference time domain
FBG	Fibre Bragg Grating
OFDR	Optical Frequency Domain Reflectometry
DAS	Distributed acoustic sensing
BOTDA	Brillouin Optical Time Domain Analysis
OFS	Optical-fibre sensing
SBAS	Small Baseline Subset
UASs	Uncrewed Aerial Systems
